# PAX8 lineage-driven T cell engaging antibody for the treatment of high-grade serous ovarian cancer

**DOI:** 10.1038/s41598-021-93992-1

**Published:** 2021-07-21

**Authors:** Emily Lee, Sarah Szvetecz, Ryan Polli, Angelo Grauel, Jayson Chen, Joyce Judge, Smita Jaiswal, Rie Maeda, Stephanie Schwartz, Bernd Voedisch, Mateusz Piksa, Chietara Japutra, Lingheswar Sadhasivam, Yiqin Wang, Ana Carrion, Sinan Isim, Jinsheng Liang, Thomas Nicholson, Hong Lei, Qing Fang, Michelle Steinkrauss, Dana Walker, Joel Wagner, Viviana Cremasco, Hui Qin Wang, Giorgio G. Galli, Brian Granda, Keith Mansfield, Quincey Simmons, Andrew Anh Nguyen, Nicole Vincent Jordan

**Affiliations:** 1 Novartis Institutes for Biomedical Research, Cambridge, MA USA; 2 NIBR Biologics Center, Novartis Institutes for Biomedical Research, Cambridge, MA USA; 3 PKS Oncology, Novartis Institutes for Biomedical Research, Cambridge, MA USA; 4 Immuno-Oncology, Novartis Institutes for Biomedical Research, Cambridge, MA USA; 5 PCS Toxicology, Novartis Institutes for Biomedical Research, East Hanover, NJ USA; 6 PCS Toxicology, Novartis Institutes for Biomedical Research, Cambridge, MA USA; 7 NIBR Biologics Center, Novartis Institutes for Biomedical Research, Basel, Switzerland; 8 Oncology, Novartis Institutes for Biomedical Research, Basel, Switzerland

**Keywords:** Biotechnology, Cancer, Drug discovery, Immunology

## Abstract

High-grade serous ovarian cancers (HGSOC) represent the most common subtype of ovarian malignancies. Due to the frequency of late-stage diagnosis and high rates of recurrence following standard of care treatments, novel therapies are needed to promote durable responses. We investigated the anti-tumor activity of CD3 T cell engaging bispecific antibodies (TCBs) directed against the PAX8 lineage-driven HGSOC tumor antigen LYPD1 and demonstrated that anti-LYPD1 TCBs induce T cell activation and promote in vivo tumor growth inhibition in LYPD1-expressing HGSOC. To selectively target LYPD1-expressing tumor cells with high expression while sparing cells with low expression, we coupled bivalent low-affinity anti-LYPD1 antigen-binding fragments (Fabs) with the anti-CD3 scFv. In contrast to the monovalent anti-LYPD1 high-affinity TCB (VHP354), the bivalent low-affinity anti-LYPD1 TCB (QZC131) demonstrated antigen density-dependent selectivity and showed tolerability in cynomolgus monkeys at the maximum dose tested of 3 mg/kg. Collectively, these data demonstrate that bivalent TCBs directed against LYPD1 have compelling efficacy and safety profiles to support its use as a treatment for high-grade serous ovarian cancers.

## Introduction

High-grade serous ovarian cancer (HGSOC) is the most aggressive gynecological malignancy^1^. Due to the high rates of recurrence following standard of care chemotherapy in combination with platinum taxanes and the late-stage presentation, the 5-year survival rate for patients diagnosed with HGSOC is roughly 30%^1,2^. Poly(adenosine 5′-disphosphate-ribose) polymerase inhibitors (PARPi) for patients with BRCA or homologous recombination deficiency mutations has improved overall survival by 30%^3^. Furthermore, early clinical trial results showed modest clinical benefit in 10–15% of patients from blockade of programmed cell death protein (PD-1) monotherapy, indicating that ovarian cancer may be amenable to certain forms of immunotherapy^4^. Despite these recent treatment advances, targeted therapies for patients with advanced HGSOC are urgently needed.

CD3 T cell engaging bispecific antibodies (TCBs) have demonstrated marked efficacy in the hematological malignancies because these molecules can broadly activate and redirect cytotoxic T cells toward tumor cells. Blinatumomab, which targets CD19, is the first bispecific T cell engager (BiTE) in clinical development with demonstrated efficacy in relapsed/refractory B-cell precursor acute lymphoblastic leukemia and non-Hodgkin lymphoma^5–7^. BiTEs and traditional CD3 bispecific antibodies targeting BCMA have similarly demonstrated 70% clinical response rates in multiple myeloma patients^8,9^. However, in solid tumors, the narrow therapeutic index has been a major challenge for the successful development of TCBs against targets such as CEA, PSMA and HER2^10–12^. Despite these challenges, TCBs targeting tumor-restricted antigens are emerging as potent forms of immunotherapy in advanced-stage solid tumors of high unmet medical need. Recent single agent clinical responses were shown by the anti-DLL3xCD3 (AMG757) in small cell lung cancer and by the anti-MUC16xCD3 (REGN4018) in advanced ovarian cancers^13,14^.

Due to the narrow therapeutic index of CD3 bispecific antibodies, it is important to identify targets with limited normal tissue expression and high tumor selectivity. Lineage-specific targets have the potential to minimize toxicity to non-tumor derived normal tissue compartments. PAX8 is a paired box transcription factor and known lineage-driver for HGSOC. Both shRNA and CRISPR knock-out (KO) screens have shown a strong PAX8 lineage dependency in HGSOC^15,16^. LYPD1 is a GPI-anchored membrane protein with medium-to-high cell surface expression in HGSOC. The expression of LYPD1 is limited to tissues of origin, such as the fallopian tube and anterior pituitary gland, making it a compelling TCB target for HGSOC^17^.

Herein, we describe the transcriptional regulation of LYPD1 expression by PAX8 in HGSOC tumor cells. Furthermore, we demonstrate the preclinical efficacy and non-human primate toxicology profile of QZC131, an anti-LYPD1 TCB with bivalent low-affinity anti-LYPD1 antigen-binding fragments (Fabs) and monovalent anti-CD3 scFv that selectively targets LYPD1^high-medium^-expressing tumor cells with high potency while sparing LYPD1^low^-expressing tumor cells. The compelling efficacy and safety profile of the bivalent low affinity anti-LYPD1 TCB directed against the PAX8 lineage-driven target LYPD1 demonstrates its therapeutic potential for the treatment of HGSOC.

## Results

### HGSOC is PAX8 dependent and LYPD1 is a PAX8 transcriptional target

**Figure 1:**
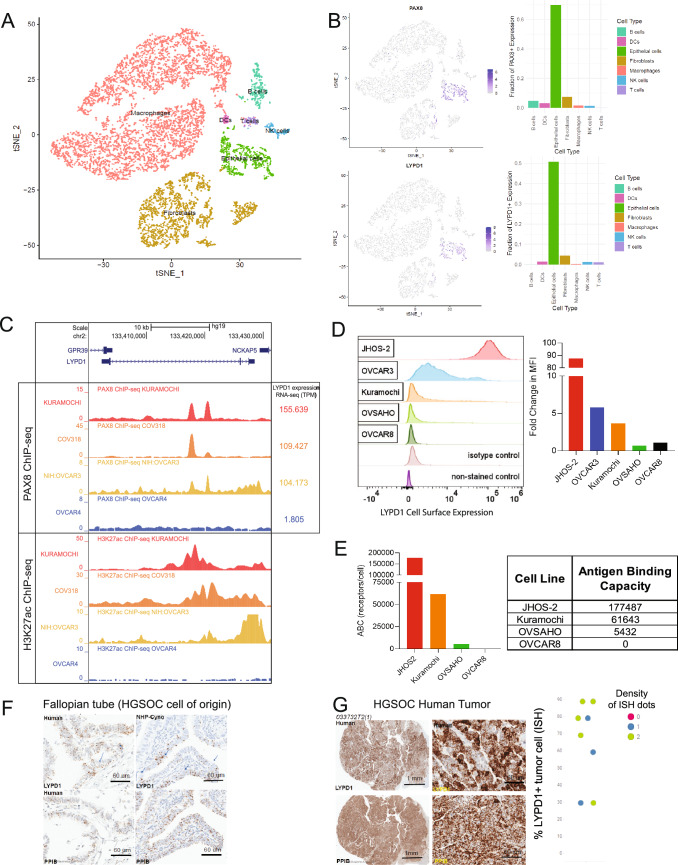
HGSOC is PAX8 dependent and LYPD1 is a PAX8 transcriptional target. (**A**) TSNE plot of 9609 single cells derived from the ascites of 11 HGSOC patients and sequenced by 10X genomics demonstrates malignant (epithelial) and non-malignant (fibroblasts, macrophages, B, T, NK-cells and DCs) cell populations. (**B**) PAX8 and LYPD1 show epithelial compartment expression in 69.8% and 50.7% of total epithelial cells sequenced. Scale bar indicates the normalized expression intensity. (**C**) ChIP-seq identifies PAX8 and H3K27Ac on an intragenic enhancer of the LYPD1 gene in the ovarian cancer cell lines (KURAMOCHI, COV318, OVCAR3) having LYPD1 mRNA expression. (**D**) LYPD1 cell surface expression is shown by FACS for the ovarian cancer cell lines JHOS2 (high), OVCAR3 (medium/high), Kuramochi (medium/low), OVSAHO (low) with no expression in the LYPD1-negative line OVCAR8. (**E**) LYPD1 antigen density levels (receptors/cell) across the ovarian cancer cell lines represented in (**D**) demonstrate the same rank-order JHOS2 (177,487 receptors/cell), Kuramochi (61,643 receptors/cell) and OVSAHO (5432 receptors/cell). (**F**) Representative RNA in situ hybridization (RNA-ISH) demonstrates moderate levels of LYPD1 RNA expression distributed across the HGSOC tissue of origin, the fallopian tube. Scale bar marks 60 μm. (**G**) Representative RNA-ISH image shows high level LYPD1 RNA expression distributed uniformly across eight HGSOC tumors. Scale bar marks 60 µ1 mm.

PAX8 is a functional oncogenic transcription factor in HGSOC. Both genome-wide shRNA knock-down (KD) and CRISPR knock-out (KO) screens have demonstrated this lineage dependency in which PAX8 KD and KO result in ovarian cancer lineage growth arrest^15,16^. Re-analysis of single-cell RNA-seq (scRNA-seq) 10X data from Izar et al., which included 9,609 high-quality individual cells from 22 ascites samples across 11 patients showed PAX8 to be expressed in 69.8% of the total malignant epithelial cell population (Fig. 1A,B and Supplementary Fig. 1A)^18^. Due to the lack of effective PAX8 therapeutic targeting strategies, we examined cell surface targets showing concordant expression with PAX8. LYPD1 is a GPI-anchored cell surface target whose expression is restricted to the malignant epithelial cell population and occurs in greater than 50.7% of the malignant cells sequenced (Fig. 1B). Conversely, known ovarian cancer targets MUC16 and MSLN were not epithelial-restricted and were expressed in both the fibroblast population (12.4% MUC16+, 53.1% MSLN+) and the malignant epithelial cell population (51.3% MUC16+, 78.5% MSLN+) (Supplementary Fig. 1B, C). Due to the relatively low recovery (7.9%) of malignant epithelial cells from the ascites of the 11 HGSOC patients, Izar et al. analyzed an additional 1297 viable malignant cells isolated on the basis of EPCAM+/CD24+ flow sorting and sequenced by full-length scRNA-seq SMART-seq2^17^. In these data, PAX8, LYPD1, MUC16 and MSLN were expressed in most malignant epithelial cells (92, 87, 97, and 95%, respectively) and still expressed in a small population of fibroblasts also captured (23, 3, 84, and 83%, respectively) (Supplementary Figs. 1D–H). LYPD1 and PAX8 demonstrated co-expression in 42.2% of the malignant cells sequenced by 10X (Supplementary Fig. 1A, J) and 81.3% of the epithelial malignant cells sequenced by SMART-seq2 (Supplementary Fig. 1I, K).

In order to understand the connection between PAX8 and LYPD1 in HGSOC, we conducted PAX8 ChIP-seq across a set of four ovarian cancer cell lines ranked by Domcke et al. among the top 17 most representative cell line models for HGSOC tumors^19^. PAX8 occupies an intragenic enhancer in the LYPD1 gene locus for LYPD1-expressing ovarian cancer cell lines OVCAR3, KURAMOCHI, and COV318 but not OVCAR4 (Fig. 1C), suggesting that PAX8 might contribute to the transcriptional regulation of LYPD1 expression.

LYPD1 is a novel GPI-anchored protein with cell surface expression confirmed across a panel of HGSOC cell lines (Fig. 1D,E), making it a tractable drug target. The antibody binding capacity, defined as the number of antibody molecules bound to the cell surface under saturating conditions, ranged from 177,487 receptors/cell for JHOS2 (LYPD1^high^ HGSOC line) to 61,643 receptors/cell for KURAMOCHI (LYPD1^medium/low^ HGSOC line) to 5432 receptors/cell for OVSAHO (LYDP1^low^ HGSOC line) (Fig. 1D,E). LYPD1 expression is largely tumor-restricted with expression limited to lineage-specific tissues of origin such as the fallopian tube, anterior pituitary gland and neurons in the prefrontal cortex of the brain, as measured by RNA-ISH (Fig. 1F and Supplementary Fig. 2A). Examination of a limited panel of tumors by RNA-ISH demonstrated expression in 100% of HGSOC resections with 75% of samples positive in greater than 50% of the tumor cells (Fig. 1G and Supplementary Fig. 2A). Using dissociated non-human primate pituitary tissue to benchmark the highest level of cell surface-expressed LYPD1 in normal tissue, we identified the OVSAHO cell line with 5432 receptors/cell as having expression most reflective of normal pituitary tissue (Fig. 1E and Supplementary Fig. 2A, B). Based upon the high tumor prevalence and the lineage restricted normal tissue profile, LYPD1 is an attractive target for the T cell-engaging therapeutic modality.

### LYPD1-directed TCBs activate T cells to induce target-dependent T cell-mediated cytotoxicity

**Figure 2:**
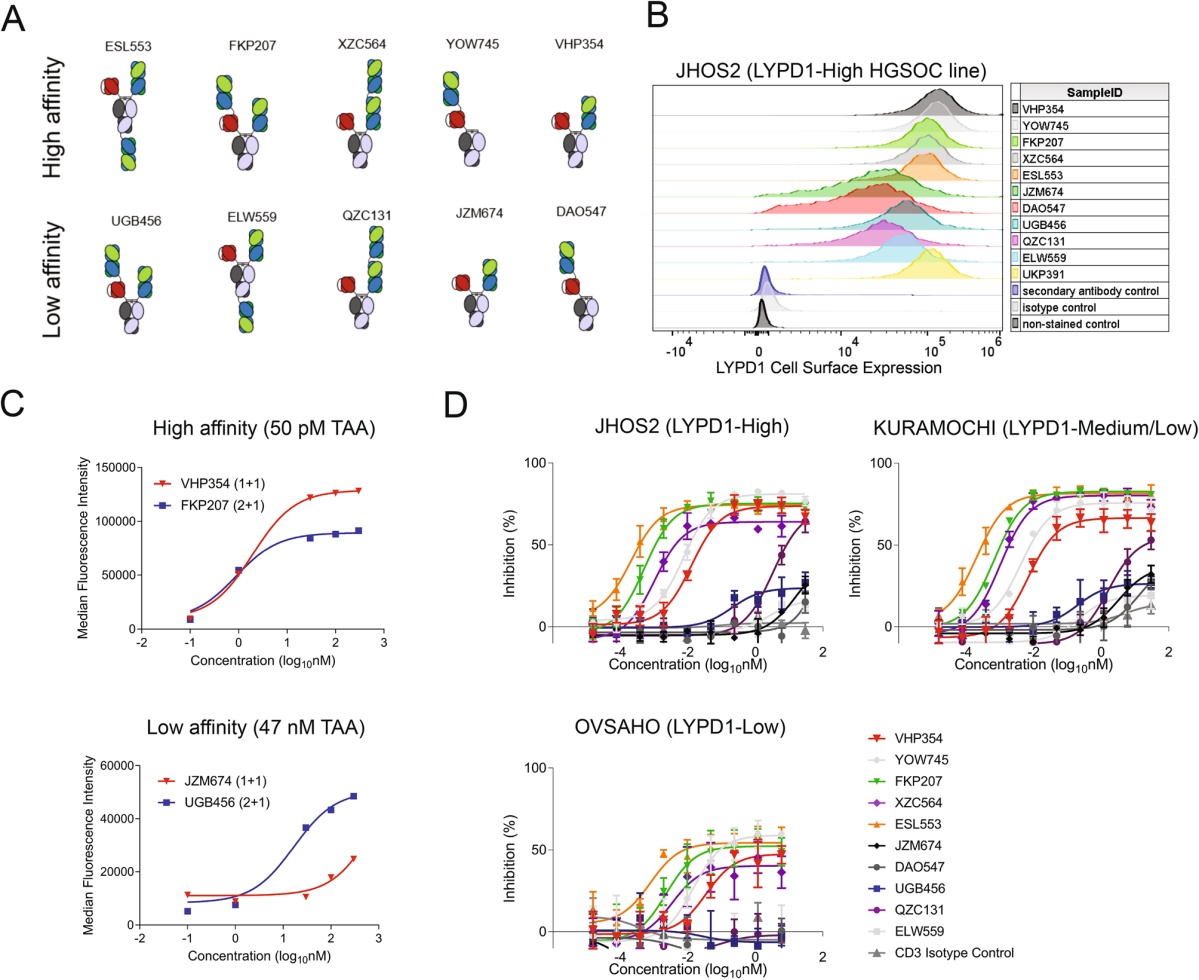
LYPD1-directed TCBs activate T cells to induce target-dependent T cell-mediated cytotoxicity. (**A**) Schematic of LYPD1-directed TCBs with two low-affinity (47 nM) bivalent anti-LYPD1 Fab arms (green) and one anti-CD3 scFv (red), termed 2 + 1 TCB, or one high affinity (50 pM) monovalent anti-LYPD1 fab arm and one anti-CD3 scFv, termed 1 + 1 TCB, across multiple TCB geometries. (**B**) FACS histograms demonstrate LYPD1-specific binding across all formats on the ovarian cancer cell line JHOS2. (**C**) FACS titration curves show dose-dependent binding to JHOS2 for LYPD1-monovalent 1 + 1 TCB (red) and LYPD1-bivalent 2 + 1 TCB (blue) with no change in the FACS EC50 for high affinity binders but 30-fold improved binding for low affinity binders. (**D**) LYPD1-directed TCBs with high affinity TAA demonstrate selective and dose-dependent induction of T cell-mediated cytotoxicity across all cell lines regardless of antigen density, while LYPD1-bivalent 2 + 1 TCBs with low affinity induce T cell-mediated cytotoxicity only in the high (JHOS2) and medium/low (KURAMOCHI) antigen density cell lines and spare the LYPD1-low expressing cell line OVSAHO. 10-point dose response curves from a starting concentration of 30 nM demonstrate activity differentials across TCBs.

The success of T cell engaging therapies is limited by the narrow therapeutic index, which can be restricted by the lack of tumor-selective cell surface expression or the affinity of the tumor antigen-targeting arm (TAA). In order to overcome these challenges, tumor selectivity can be enhanced using avidity-driven TAAs. Therefore, we compared the efficacy of LYPD1-directed T cell-engaging antibodies (TCBs) with bivalent low-affinity (47 nM) anti-LYPD1 Fabs and 16 nM anti-CD3 scFv (2 + 1 TCB) to the monovalent high affinity (50 pM) anti-LYPD1 Fab and 16 nM anti-CD3 scFv (1 + 1 TCB) across multiple geometries (Fig. 2A). Both the 1 + 1 and 2 + 1 anti-LYPD1 TCBs specifically bound to LYPD1 expressing cell lines JHOS2, OVCAR3, KURAMOCHI and OVSAHO (Fig. 2B and Supplementary Fig. 3A). For the high affinity molecules, we did not observe a change in the FACS EC50 of cellular binding when comparing 1 + 1 to 2 + 1 TCB formats, despite slightly higher maximum MFI values (Fig. 2C). However, the low affinity anti-LYPD1 TCBs in 2 + 1 format improved cellular binding by greater than 30-fold when compared to the 1 + 1 low affinity format (Fig. 2C). The avidity-driven effects of the 2 + 1 TCB format were also demonstrated biochemically by surface plasmon resonance (SPR), in which slower K_off_ rates were measured for the 2 + 1 TCB format but not for the 1 + 1 TCB format as LYPD1 antigen densities increased (Supplementary Fig. 3B).

The cytotoxic potency of anti-LYPD1 TCBs across 2 + 1 and 1 + 1 formats was evaluated across the same panel of ovarian cell lines expressing a range of LYPD1 antigen densities. The high affinity anti-LYPD1 TCBs in 1 + 1 (VHP354 and YOW745) and 2 + 1 (FKP207, XZC564 and ESL553) formats induced LYPD1-dependent T cell-mediated cytotoxicity across all cell lines, regardless of antigen density levels. Half-maximal inhibitory concentrations (IC50s) ranged from 0.8 pM to 33.2 pM for VHP354 (1 + 1) and from 0.5 pM to 2.4 pM for FKP207 (2 + 1) (Fig. 2D and Supplementary Fig. 3C). Conversely, the low affinity anti-LYPD1 TCBs in 1 + 1 and 2 + 1 formats only induced potent T cell-mediated cytotoxicity in the LYPD1^medium^ and LYPD1^high^ -antigen density cell lines while sparing the LYPD1^low^-expressing cell line OVSAHO (Fig. 2D and Supplementary Fig. 3C). Among all of the low affinity 2 + 1 molecules, the anti-LYPD1 TCB with stacked tandem fab geometry (QZC131) uniquely induced cytotoxicity in each of the LYPD1^high^ and LYPD1^medium^-expressing cell lines, including JHOS2, OVCAR3, KURAMOCHI, but did not induce cytotoxicity in the LYPD1^low^-expressing cell line OVSAHO or the LYPD1^negative^ cell line OVCAR8 (Fig. 2D and Supplementary Fig. 3C). Additionally, OVCAR8 engineered to overexpress LYPD1 at 2.6-fold above background demonstrated no change in cellular growth characteristics but showed LYPD1-dependent TCB-mediated cytotoxicity with IC50 values similar to that of endogenously-expressing HGSOC cell lines (Supplementary Fig. 3D,E). These results indicate that high affinity anti-LYPD1 TCBs are unable to distinguish among antigen densities, while the bivalent low affinity anti-LYPD1 TCBs with stacked tandem fab geometry is able to distinguish among antigen densities.

### In vitro and in vivo cytokine response in genetically engineered mice is affinity-driven

**Figure 3:**
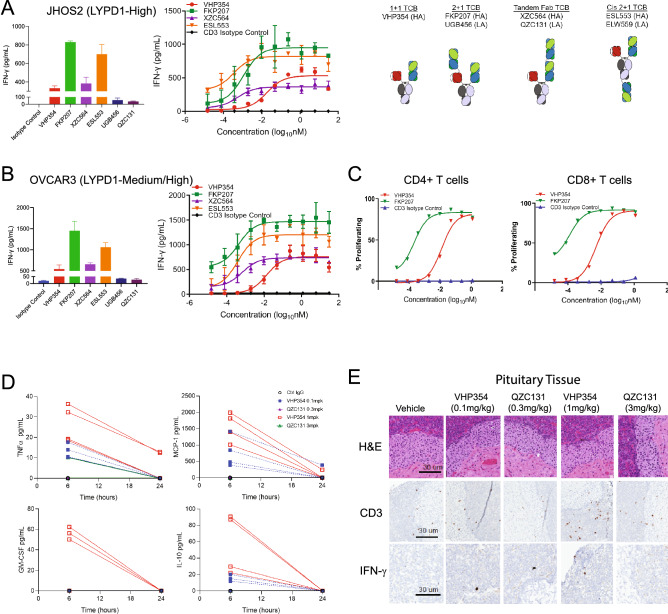
In vitro and in vivo cytokine response in genetically engineered mice is affinity-driven. Cytokine release assays by MSD demonstrate dose-dependent IFN-γ production in the (**A**) high LYPD1-expressing cell line JHOS2 and (**B**) medium/high LYPD1-expressing cell line OVCAR3 following 48 h treatment with the LYPD1-directed TCBs. High affinity LYPD1-bivalent 2 + 1 formats FKP207 (green) and ESL553 (orange) show 2–3 fold higher IFN-γ levels compared to matched LYPD1-monovalent 1 + 1 TCB VHP354 (red), while VHP354 (red) and XZC564 (purple) demonstrate 10–20 fold higher IFN-γ levels compared to low affinity TCBs QZC131 (dark purple) and UGB456 (dark blue). (**C**) T cell proliferation assays in the LYPD1-expressing cell line OVCAR3 following treatment with LYPD1-directed TCBs reveal a 50-fold difference in both CD4 + and CD8 + T cell proliferation between LYPD1-bivalent 2 + 1 FKP207 (green) and LYPD1 monovalent 1 + 1 VHP354 (red) formats. (**D**) Magnitudes of change for TNFα, MCP1, IL10 and GM-CSF from CD3 human transgenic mice were greatest at 6 h post dose compared with other study time-points and decreased at 24 h post dose with VHP354 demonstrating a trend toward enhanced cytokines TNFα, MCP1, IL10 and GM-CSF at both doses. (**E**) Histopathology shows no evidence of tissue destruction, while CD3 IHC and IFN-γ RNA-ISH demonstrate increased CD3 and IFN-γ levels (brown stain). Scale bar 30 um.

TCBs are hindered by the systemic induction of cytokine release syndrome (CRS). However, cytokine release is critical for T cell-mediated cytotoxicity and T cell proliferation, and the in vitro interferon-γ (IFN-γ) levels can inform both our understanding of potency and in vivo tolerability. Therefore, in vitro T cell activation was measured as a function of IFN-γ cytokine release and subsequent T cell proliferation. LYPD1-directed TCBs with high affinity Fabs induced IFN-γ production in both the LYPD1^high^ and LYPD1^medium^-expressing cell lines JHOS2, KURAMOCHI and OVCAR3 but did not induce IFN-γ in the LYPD1^negative^ line OVCAR8 (Fig. 3A,B and Supplementary Figs. 4A,B). While the maximal IFN-γ release was cell-line dependent, the IFN-γ fold change at saturating concentrations across ovarian cancer cell lines treated with LYPD1-directed TCBs followed the same rank order as the FACS-binding EC50s. (Fig. 3A,B and Supplementary Fig. 4B). The high affinity 2 + 1 anti-LYPD1 TCBs, FKP207 and ESL553, showed 2–3 fold higher IFN-γ levels compared to the 1 + 1 anti-LYPD1 TCB VHP354. Meanwhile, VHP354 and XZC564 demonstrated 10–20 fold higher IFN-γ levels compared to the 2 + 1 low affinity anti-LYPD1 TCBs, QZC131 and UGB456 (Fig. 3A,B and Supplementary Fig. 4A,B). T cell proliferation assays in the LYPD1^high/medium^-expressing cell line OVCAR3 following treatment with LYPD1-directed TCBs revealed a 50-fold difference in both the CD4 + and CD8 + T cell proliferation for the 2 + 1 high affinity anti-LYPD1 TCB compared to the 1 + 1 anti-LYPD1 TCB (Fig. 3C). Together, these results demonstrate that T cell activation and proliferation is LYPD1-dependent, and while the magnitude of cytokine release is cell line-dependent, the kinetics are both avidity- and affinity-driven.

Since LYPD1 shares the same extracellular domain sequence identity and tissue localization in both human and mouse species (Supplementary Fig. 2A), we evaluated in vivo tolerability of LYPD1-directed TCBs in the human transgenic (HuT) CD3 mouse model, which was developed by replacing the mouse sequence with the human sequence for the SP34 epitope on the CD3ε chain (Supplementary Fig. 4C). Doses of the bivalent low affinity anti-LYPD1 TCB QZC131 and the monovalent high affinity anti-LYPD1 TCB VHP354 were determined based upon the IC50 values from in vitro redirected T-cell cytotoxicity (RTCC) experiments. Four mice from each group received 0.1 mg/kg VHP354, 1 mg/kg VHP345, 0.3 mg/kg QZC131, 3 mg/kg QZC131, or isotype control as a single intravenous bolus injection. Seven days following administration of VHP354 at 0.1 or 1 mg/kg doses and QZC131 at 0.3 and 3 mg/kg doses, animals were euthanized, and tissues were harvested. Relative to isotype control dosed animals, the cytokines IFN-γ, IL6 and IL-1β were unchanged with undetectable levels of IL-1β (Supplementary Fig. 4D). Magnitudes of change for TNFα, MCP1, IL10 and GM-CSF were greatest at 6 hours (h) post-dose compared with other time-points and decreased at 24 h post-dose (Fig. 3D). While there was a trend toward enhanced cytokines TNFα, MCP1, IL10 and GM-CSF with both doses of VHP354, the magnitudes of change were not notable (Fig. 3D). Body weight and the histopathology of LYPD1-expressing tissues, including pituitary, pancreas, testes, and brain, were unchanged (Supplementary Fig. 4E). Despite absence of histology and cytokine differences, the pituitary but not the pancreas, colon or brain, demonstrated CD3 + infiltrates and IFN-γ expression at 0.1 and 1 mg/kg VHP354 and at 1 and 3 mg/kg QZC131 without changes in IL6, TNFα or IL-1β (Fig. 3E and Supplementary Fig. 4F). These data indicate VHP354 and QZC131 can activate T cells in vivo without inducing systemic cytokine response or histopathology in HuT CD3 mouse models.

### Toxicology study of VHP354 and QZC131 in cynomolgus monkeys demonstrates systemic cytokine release syndrome is affinity-driven

To determine the safety and tolerability of the monovalent high affinity anti-LYPD1 TCB VHP354 and bivalent low affinity anti-LYPD1 TCB QZC131 and to characterize the pharmacokinetics of these molecules, we conducted a single-dose toxicology study in cynomolgus monkeys. Doses of QZC131 and VHP354 were determined based upon in vitro RTCC experiments and historical TCB data in monkeys. One monkey from each group received a sentinel dose of 0.1 mg/kg VHP354 or 0.3 mg/kg QZC131. Following the sentinel dose, two monkeys from each group received 1 and 3 mg/kg, respectively, as a single intravenous bolus injection (Supplementary Table 1). Following the 7-day observation period, animals were euthanized on day 8, and tissues were examined for microscopic findings. QZC131 was well-tolerated, and all animals survived to the time of scheduled necropsy. However, animal P201, which was administered the sentinel dose of 0.1 mg/kg VHP354 was euthanized on day 2 due to overtly decreased general activity, lack of appetite, hunched posture, and pale pink to blueish mucous membrane. However, no changes were observed in the histopathology of LYPD1-expressing tissues, including pituitary, pancreas, liver and colon, from all dosed animals including animal P201 (Supplementary Fig. 5A). The clinical pathology effects of the animal dosed with VHP354 were supportive of inflammation and correlated with clinical observations of markedly increased C-reactive protein (CRP) and mildly decreased albumin concentrations (Supplementary Table 2). Animals dosed with QZC131 survived until scheduled euthanasia and showed minor increases in CRP concentrations without dose response as well as minimal decreases in albumin concentrations day 2 post-dose. By day 8 post-dose, all values returned to pre-dose levels (Supplementary Table 2).

**Figure 4:**
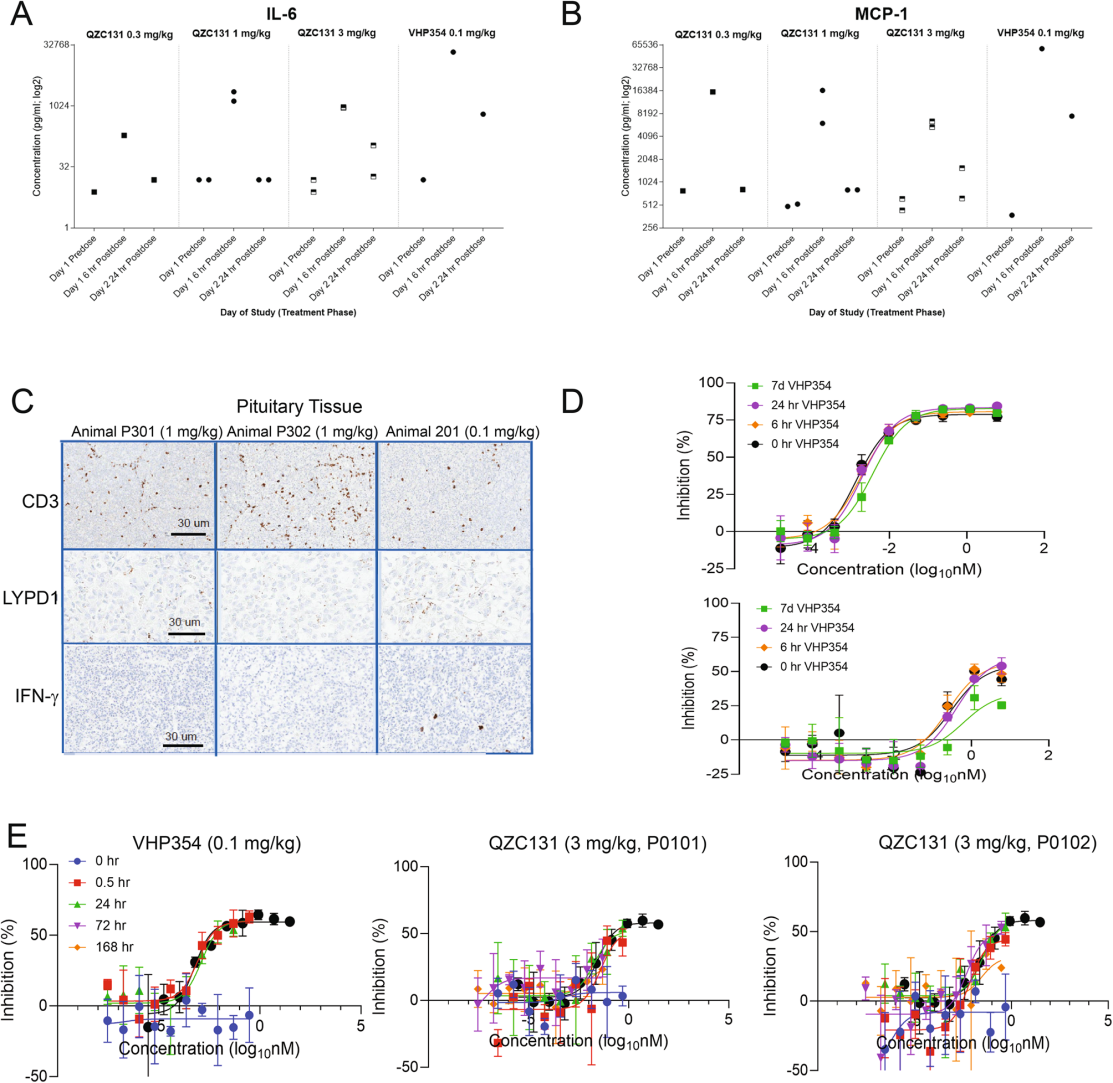
Toxicology study of VHP354 and QZC131 in cynomolgus monkeys demonstrates systemic cytokine release syndrome to be affinity-driven. (**A**,**B**) Treatment of cynomolgus monkeys with LYPD1-directed TCBs VHP354 and QZC131 showed increased serum IL-6 and MCP-1 at 6 h post-dose with partial to complete resolution by 24 h post-dose. Cytokine changes by greater than 2-fold were considered meaningful. (**C**) IHC of CD3 + infiltrates showed increased CD3 + cells in the pituitary tissue across all dose groups with a correlative increase in IFN-γ levels in the pituitary of all dosed animals. Scale bar represents 30 µm. (**D**) RTCC assays with LYPD1-high expressing cells U251 demonstrate selective and dose-dependent induction of T cell-mediated cytotoxicity for VHP354 and QZC131 following 0, 6, 24, and 168 h incubation in cynomolgus serum at 37 °C. 10-point dose response curves from a starting concentration of 30 nM of VHP354 or QZC131 at 0 h (black), 6 h (orange), 24 h (purple), and 168 h (green) demonstrate comparable activity across time-points. (**E**) RTCC assays with DMS273-LYPD1 expressing cells demonstrate selective and dose-dependent induction of T cell-mediated cytotoxicity from animal P0201 serum for VHP354 (0.1 mg/kg) at 0.5 and 24 h post-dose and from animals P0101 and P0102 (3 mg/kg) for QZC131 (3 mg/kg) at 0.5, 24, 72 and 168 h post-dose. 10-point dose response curves from a starting concentration of 30 nM of VHP354 or QZC131 serum spike-in control (black), 0.5 h (red), 24 h (green), 72 h (purple) and 168 h (orange) demonstrate comparable activity across time-points.

Magnitudes of change for cytokines were greatest at 6 h post-dose and decreased by 24 h post-dose. The specific analytes assessed were IL-1β, IL-1RA, IL-2, IL-5, IL-6, IL-10, IL-12p70, IL-17, IFN-γ, IP-10, MCP-1 and TNFα. Changes of 2-fold or greater than pre-dose values were considered meaningful. VHP354 and QZC131 induced increased in serum IL-6, MCP-1, IL-1RA, IFN-γ, IL-17 and 1L-12p70 at 6 h post-dose (Fig. 4A,B, Supplementary Fig. 5B and Supplementary Table 3). Additionally, animals dosed with 0.3, 1 and 3 mg/kg QZC131 or 0.1 mg/kg VHP354 showed increases in IL-2 and IL-10. All of these changes showed partial to complete resolution by 24 h post-dose. Notably, in the animal given VHP354 (0.1 mg/kg) and euthanized in moribund condition on day 2, the magnitudes of change in IL-6, MCP-1, IL-1RA and IFN-γ were greater at 6 h post-dose compared to all other study animals and time-points (Fig. 4A,B, Supplementary Fig. 5B and Supplementary Table 3). Although there was no histological evidence of LYPD1-expressing normal tissue destruction in the brain, pituitary or pancreas, moderate increases in CD3 positive cell infiltrates were observed in both the pituitary and pancreas (Fig. 4C and Supplementary Fig. 5C). The increased CD3 positive cells correlated with increased IFN-γ in the pituitary of all dosed animals (Fig. 4C). The clinical findings following cynomolgus monkey treatment of VHP354 were indicative of severe CRS, while there was no evidence of severe CRS following treatment with QZC131.

A noncompartmental pharmacokinetic (PK) analysis was conducted using Phoenix WinNonlin® with the PK results from the NHP toxicology study (Supplementary Table 4)^20^. QZC131 concentrations declined in a bi-exponential manner and demonstrated linear pharmacokinetics, with dose normalized area under the curve (AUC) being similar between animals and dose groups (Supplementary Fig. 5D). QZC131 clearance was faster than expected when compared to known values for humanized IgGs in monkeys, which range from 0.1 to 0.5 mL/h/kg^19^. The terminal half-life of QZC131 was similar between animals and across dose groups, averaging 56.4 ± 9.23 h. Although the clearance and half-life of QZC131 was unexpected, steady state volume of distribution (V_ss_) was approximately that of serum volume for a cynomolgus monkey, 40.5 mL/kg, consistent with prior observations of IgG-based therapies (Supplementary Fig. 5D and Supplementary Table 4). Safety concerns due to clinical signs of CRS for VHP354 prevented escalation of doses beyond 0.1 mg/kg. VHP354 demonstrated rapid clearance of 1.44 mL/h/kg, 1.8-fold greater than QZC131 (Supplementary Fig. 5D and Supplementary Table 4). Considering much of the terminal phase of VHP354 was lost due to euthanasia, calculation of average clearance (CL) for QZC131 and VHP354 out to 24 h determined both molecules exhibit similar values: 2.49 versus 2.32 mL/h/kg, respectively (Supplementary Fig. 5D and Supplementary Table 4).

Despite the rapid PK clearance of the anti-LYPD1 TCBs, stability and activity was confirmed out to 168 h post-dose. The serum stability of VHP354 and QZC131 was measured by incubating anti-LYPD1 TCBs in serum from cynomolgus monkeys over a 7-day time-course and measuring T cell-mediated cytotoxic activity at 0, 6, 24 and 168 h. T cell-mediated cytotoxicity for both VHP354 and QZC131 was retained to 168 h without inducing cytotoxicity in the LYPD1^negative^ cell line OVCAR8, indicating TCB stability (Fig. 4D and Supplementary Fig. 5E). Residual serum samples from the bioanalytical blood collections were used for confirming activity of the molecule via in vitro RTCC assay in the engineered LYPD1-expressing cell line DMS273-LYPD1-OE (Supplementary Fig. 5F and Supplementary Fig. 6A). The time-points used for analyses were pre-dose and approximately 0.5, 24, 72, and 168 h post-dose, when available. Both QZC131 and VHP354 induced LYPD1-specific cytotoxicity in DMS273-LYPD1-OE across all time-points tested (Fig. 4E). In contrast, QZC131 and VHP354 did not induce cytotoxicity in the LYPD1^negative^-expressing cell line OVCAR8 (Supplementary Fig. 5G). QZC131 and VHP354 showed comparable initial activity in serum at both the pre-dose and post-dose time-points tested, as determined by IC50 and maximum lysis values of T cell-mediated cytotoxicity assays (Fig. 4E and Supplementary Table 5). Notably, there was more variability between QZC131 (3 mg/kg) dosed animals (P101 and P102) at 72 and 168 h (Fig. 4E and Supplementary Table 5). In contrast, VHP354 (0.1 mg/kg) showed no change in serum T cell-mediated cytotoxicity at 24 h post-dose, but the full time-course was incomplete due to unscheduled animal euthanasia. Together these results demonstrate that the LYPD1-directed TCBs VHP354 and QZC131 are stable and can activate T cells in vivo for up to 168 h post-dose. VHP354 but not QZC131 induced systemic CRS in an affinity-driven manner without evidence of target-mediated tissue destruction in cynomolgus monkeys.

### VHP354 and QZC131 activate T cells and reduce tumor burden in an endogenous LYPD1-expressing xenograft tumor model in a dose-dependent manner

**Figure 5:**
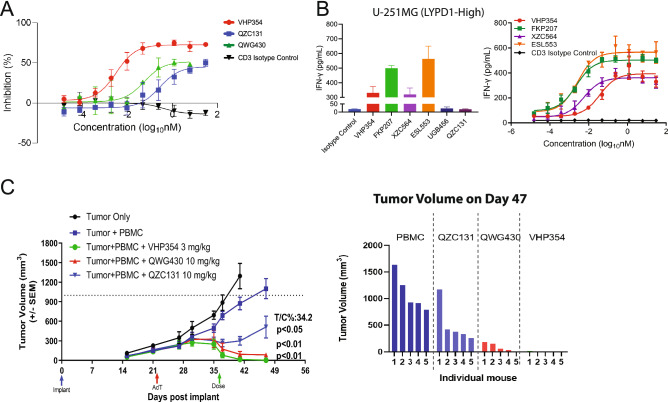
VHP354 and QZC131 activate T cells and reduce tumor burden in an endogenous LYPD1-expressing xenograft tumor model in a dose-dependent manner. (**A**) In vitro RTCC assays with LYPD1-high expressing cells U251 demonstrate selective and dose-dependent induction of T cell-mediated cytotoxicity for VHP354 (red), QWG430 (green) and QZC131 (blue). 10-point dose response curves from a starting concentration of 100 nM of VHP354 (red), QWG430 (green) or QZC131 (blue) demonstrate differential activity across time-points. (**B**) Cytokine release assays by MSD demonstrate dose-dependent IFN-γ production in the high LYPD1-expressing cell line U251 following 48 h treatment with the LYPD1-directed TCBs FKP207 (green), ESL553 (orange), VHP354 (red), XZC564 (purple), QZC131 (dark purple) and UGB456 (dark blue). (**C**) In vivo xenograft AdT model demonstrates LYPD1-directed TCBs VHP354 QWG430 and QZC131 induce tumor growth inhibition. VHP354 and QWG430 induced tumor regressions in all animals tested, while QZC131 in inhibited tumor growth in 4/5 animals tested.

To investigate the antitumor efficacy of bivalent anti-LYPD1 TCBs in comparison to monovalent anti-LYPD1 TCBs, we used the immunodeficient NOD SCID gamma mouse (NSG) with human PBMCs delivered by adoptive transfer 14 days prior to implantation of the LYPD1-expressing tumor cells. LYPD1 expression, in vitro T cell-mediated cytotoxicity and IFN-γ cytokine release were confirmed for the xenograft cell line model U251 and demonstrated dose-dependent T cell-mediated cytotoxicity and activation in alignment with previous in vitro RTCC experiments (Fig. 5A, B and Supplementary Fig. 6A). Ablation of LYPD1 with CRISPR-CAS9 demonstrated LYPD1-selective induction of T cell-mediated cytotoxicity in U251 cells (Supplementary Fig. 6B,C). All mice in these studies were given one dose of VHP354 at 0.03, 0.3, or 3 mg/kg, QWG430 at 0.03, 0.3, 3, or 10 mg/kg, or QZC131 at 0.3, 3 or 10 mg/kg without body weight loss (Supplementary Fig. 6D). VHP354 induced tumor regressions at 3 and 0.3 mg/kg in all animals tested (Fig. 5C and Supplementary Fig. 6E). The bivalent low affinity anti-LYPD1 TCB with 5 nM CD3-targeting arm QWG430 induced dose-dependent tumor regressions at 10 and 3 mg/kg (Fig. 5C and Supplementary Fig. 6F), while the bivalent low affinity anti-LYPD1 TCB with 16 nM CD3-targeting arm QZC131 was less active but still demonstrated dose-dependent tumor growth inhibition at 10 and 3 mg/kg (Fig. 5C and Supplementary Fig. 6G). The in vivo xenograft adoptive transfer (AdT) model demonstrates that both VHP354 and QZC131 can broadly activate and redirect cytotoxic T cells toward tumor cells to induce human T cell-mediated cytotoxicity in vivo in an LYPD1- and dose-dependent manner. While QZC131 is less potent than VHP354, it demonstrates a better tolerability profile in cynomolgus monkeys, and therefore, QZC131 has therapeutic potential as a novel stand-alone therapy or in combination with checkpoint blockade to promote durable responses in HGSOC.

## Discussion

High-grade serous ovarian cancer (HGSOC) is an aggressive malignancy with poor prognosis and is genetically defined by high copy number alterations, near universal TP53 mutation and low somatic mutation rates in protein-coding regions^20^. Recent studies from mouse models and human patients support the fallopian tube epithelium (FTE), largely comprised of PAX8-positive secretory cells, as the tumor cell of origin^21,22^. Single-cell RNA sequencing of normal FTE cells further stratified the secretory cells of the FTE into four subtypes and confirmed expression of LYPD1 in the differentiated subtype^23^. Using ChIP-seq across a panel of HGSOC cell lines, we demonstrated that LYPD1 is a PAX8 lineage-driven tumor antigen and tractable cell-surface drug target in HGSOC. Of the 47 ovarian cancer cell lines defined by the Cancer Cell Line Encyclopedia (CCLE), the cell lines selected herein were ranked by Domcke et al. among the most representative cell line models for HGSOC tumors^19^. Since these HGSOC cell lines were derived from ascitic fluid or peritoneal deposits nearly 20 years ago and have been in passage for a considerable time, we cannot draw a direct connection between these cell line models and the FTE cell of origin^19,24^. In order to study the role of PAX8 and LYPD1 with respect to the FTE cell of origin, future studies should examine PAX8 and LYPD1 expression profiles and mechanisms of transcriptional regulation in primary FTE cells engineered to overexpress mutant TP53^24,25^.

Herein, we described the preclinical efficacy and non-human primate toxicology profile of the monovalent high-affinity and bivalent low-affinity anti-LYPD1 TCBs that selectively target tumor cells expressing the PAX8 lineage-driven cell surface antigen LYPD1 in HGSOC. Both monovalent high affinity and bivalent low affinity anti-LYPD1 TCBs induced potent cytotoxicity in vitro and in vivo against LYPD1-expressing cells. However, the LYPD1-directed bivalent low affinity TCB demonstrated compelling safety profiles in cynomolgus monkeys and showed selective targeting of LYPD1^high^ and LYPD1^medium^-expressing tumor cells, supporting anti-LYPD1 TCBs for treatment of advanced stage HGSOC.

Previous studies demonstrated avidity-driven binding of the bivalent anti-HER2/CD3 TCB, which showed 10-fold enhanced binding to HER2-overexpressing cells with low binding to the HER2^low^-expressing cells^12^. For the HER2 TCB, the optimal affinity range was between Kd 25 and 50 nM, and molecules with lower affinity lost much of their activity^12^. Conversely, molecules with higher affinity failed to gain selectivity. While the affinity range is likely target dependent and dictated by multiple variables, such as copy number, tumor-to-normal antigen density differentials and the biology of internalization, the LYPD1-directed bivalent TCBs which showed selective binding to LYPD1^high^ and LYPD1^medium^-expressing cells had a Kd of 47 nM, suggesting a similar optimal affinity range for LYPD1 TAAs. To fully understand the optimal affinity range for promoting affinity-driven tumor selectivity of anti-LYPD1 TCBs, future work should explore anti-LYPD1 Fabs that span a broader affinity range and epitope diversity, since both of these parameters are predicted to influence avidity-driven selectivity and to improve overall potency.

Cytokine release syndrome is a frequent and serious side effect of the T cell-engaging modalities, CD3 bispecific and CAR T cell therapies. Therefore, we examined predictive cytokines from the serum of genetically engineered CD3 HuT mice and cynomolgus monkeys treated with anti-LYPD1 TCBs. While non-tumor bearing CD3 HuT mice showed no evidence of systemic cytokine response or histopathology resultant from inflammation, cynomolgus monkeys treated with the high affinity monovalent anti-LYPD1 TCB VHP354 showed marked increases in IL6 levels indicative of CRS. Additionally, the IL6 cytokine levels were moderately increased in cynomolgus monkeys dosed with 3 mg/kg of the bivalent low affinity anti-LYPD1 TCB QZC131, but these levels were not high enough to induce systemic cytokine response. The discordant cytokine results between CD3 HuT and cynomolgus monkey studies suggests that rodent species are not the best proxy for cytokine release in non-human primates for anti-LYPD1 TCBs, but are useful for assessing on-target mechanism of action through measuring CD3 + infiltrates. Despite CD3 infiltration into LYPD1-expressing tissues following treatment, one confounding outcome of the NHP study was the lack of tissue damage in cynomolgus monkeys treated with the high affinity anti-LYPD1 TCB VHP354, which showed CRS and resulted in early animal euthanasia. Animal euthanasia occurred at 36 h post-dose, which is of sufficient duration for the onset of target-mediated tissue damage. Future evaluation of the safety profiles of anti-LYPD1 TCBs would benefit from a larger animal sample set to have a better understanding of the relationship between CRS and tissue pathology.

The narrow therapeutic index of T cell bispecific antibodies has been a major challenge for the successful development of TCBs against solid tumor targets such as CEA, PSMA and HER2^10–12^. Recent single agent clinical responses demonstrated by the anti-DLL3xCD3 (AMG757) in small cell lung cancer and by the anti-MUC16xCD3 (REGN4018) in advanced ovarian cancers have demonstrated clinical proof-of-concept for this modality in targets with tumor-restricted expression profiles similar to LYPD1^13,14^. Widespread use of immune checkpoint inhibitors has opened up the possibilities of combination therapy of TCBs with anti-PD-1/PD-L1 inhibitors in patients. Preclinical in vivo efficacy experiments for both the anti-DLL3xCD3 (AMG757) and anti-MUC16xCD3 (REGN4018) demonstrated enhanced anti-tumor efficacy in combination with blockade of the PD-1 pathway, indicating that PD-1 blockade could be a relevant future combinatorial approach. While the monovalent high affinity anti-LYPD1 TCB, VHP354 showed tumor regressions down to 0.3 mg/kg, the in vivo potency of the bivalent low affinity anti-LYPD1 TCB, QZC131 showed tumor growth inhibition at 10-fold higher doses of 3 mg/kg. Although having lower potency, QZC131 demonstrates a better tolerability profile in cynomolgus monkeys, and therefore, has better therapeutic potential as a novel stand-alone or combination treatment strategy with checkpoint blockade for HGSOC. The presented efficacy and safety data of the bivalent low affinity anti-LYPD1 TCB QZC131 directed against the PAX8 lineage-driven target LYPD1 supports its therapeutic potential and merits further evaluation as a treatment for advanced high-grade serous ovarian cancer.

## Methods

### Isolation of PBMCs from healthy human donor leukopak

Peripheral blood mononuclear cells (PBMCs) from healthy human Donor D186026 with informed consent were isolated from a leukopak (Hemacare #PB001F-2). Briefly, two volumes of PBS were mixed with the leukopak cell suspension, and this mixture was distributed to 50 mL conical tubes, 30 mL per tube. The diluted cell suspension was carefully overlayed with 15 mL of Ficoll reagent (GE Healthcare #17-1440-03) to maintain separation of cells and Ficoll phases. A density gradient was created by centrifugation at 400×*g* for 30 min with no brake. The buffy coat interphase was carefully collected, pooled to fresh tubes, and washed twice with MACS buffer containing BSA (Miltenyi #130-091-222, #130-091-376). Cells were counted, resuspended to 1 × 10^8^ cells per mL in Cryostor10 (STEMCELL Technologies # 07930) cryopreservation media, aliquoted to vials at 1 mL each, frozen slowly to − 80 °C overnight, and then transferred to liquid nitrogen vapor phase for long-term storage the following day.

### Isolation and expansion of pan T cells from frozen PBMCs

T cell medium was prepared with RPMI 1640 (gibco # 11875-085), 10% heat-inactivated fetal bovine serum (HI-FBS; Seradigm # 1500-500), 2 mM L-glutamine (gibco # 25,030–081), 1X Penicillin–Streptomycin (gibco # 15140-122), 1X MEM Non-Essential Amino Acids Solution (gibco # 11140-050), 1 mM sodium pyruvate (gibco # 11360-070), 10 mM HEPES buffer (gibco # 15630-080), 55 µM 2-mercaptoethanol (gibco # 21985-023), and filtered through a 0.22 µm PES filter (Millipore # S2GPU05RE). Freshly-isolated T cells were resuspended to a density of 0.5 × 10^6^ cells per mL in T cell medium and cultured at a 3:1 ratio of Dynabeads (gibco # 11161D) to T cells. T cell density was maintained at 0.5 × 10^6^ cells per mL every other day for 10 days. Aliquots of 25 × 10^6^ T cells in 1 mL of FBS with 10% DMSO (Sigma D2438) were made in cryovials and frozen slowly to − 80 °C overnight. Aliquots were subsequently transferred to liquid nitrogen vapor phase for long-term storage.

### FACS

Tumor cell lines were harvested with Accutase (Sigma # A6964) and added to a 96-well U-bottom plate (Corning # 3799) at a density of 0.1 × 10^6^ cells per well. Cells were washed with PBS prior to a 5 min room temperature incubation with human Fc Block (BioLegend # 422302) which was diluted 1:50 in FACS Buffer (autoMACS Running Buffer; Miltenyi Biotec # 130-091-221). All bispecific and monoclonal antibodies were diluted to 300 nM in FACS Buffer. The UKP391 IgG1 monoclonal antibody with 50 pM anti-LYPD1 binding affinity was included as a positive control and an isotype control was included. Bispecific antibodies were added at a top concentration of 300 nM with 4 1:3 serial dilutions. Antibodies were incubated at 4 °C for 30 min. Two PBS washes were performed followed by a 10 min 4 °C incubation with eFluor780 viability dye (eBioscience # 65-0863-18) diluted 1:1000 in PBS. Two FACS Buffer washes were performed followed by addition of Alexa Fluor 647 anti-human IgG secondary antibody (Jackson ImmunoResearch # 109-606-098) diluted 1:250 in FACS Buffer and incubated for 30 min at 4 °C. Two FACS Buffer washes were performed. Data was acquired on the Attune NxT Flow Cytometer (Thermo Fisher Scientific). Data was analyzed using FlowJo software (v10). GraphPad Prism (version 8.1.2) was used to plot the data with a hyperbola non-linear fit curve.

### Redirected T cell cytotoxicity assays (RTCC)

Tumor cell lines were resuspended in T cell media, plated into white 384-well clear-bottom plates (Corning # 3765) at a seeding density of 2 × 10^3^ cells in 40 µL per well and allowed to adhere overnight. Nine 1:5 serial dilutions of the bispecific antibodies and isotype control bispecific were prepared in triplicate in a deep-well plate (Greiner Bio-One # 781,271) and stamped onto cells by the Beckman Coulter i7. Expanded T cells were thawed and added at an effector:tumor ratio of 2:1 bringing the volume to 50 µL and the top bispecific antibody concentration to 30 nM. Assay plates were incubated at 37 °C with 5% CO_2_ for 48 h. Assay plates and Bright-Glo luciferase substrate reagent (Promega # E26413) were brought to room temperature. 25 µL of Bright-Glo was added to each well and shaken for 10 min at room temperature. Luciferase signals were read using the EnVision 2102 Multi-mode plate reader.

#### For RTCC on residual serum samples

Serum samples received from the testing facility (Covance study #8416938) were stored at ≤ − 70 °C until analysis. These samples represented blood collections at dosing phase day 1 pre-dose and approximately 0.5, 24, 72, and 168 h post-dose. *For RTCC stability assays.* The bispecific antibodies were diluted in cyno serum to a concentration equivalent to 1 mg/kg and incubated at 37 °C for 7 days, 24 h, and 6 h. Tumor cell lines were resuspended in T cell media, plated into white 384-well clear-bottom plates (Corning # 3765) at a seeding density of 2 × 10^3^ cells in 25 µL per well, and allowed to adhere overnight. On the day of antibody addition, the 0 h bispecific antibody dilution was prepared in the same manner as with the other time points.

### Cytokine assays

Tumor cell lines were harvested in T cell media and seeded into a 96-well plate (Corning # 3903) at a density of 5 × 10^3^ cells in 50 µL per well. Cells were allowed to adhere overnight at 37 °C with 5% CO_2_. Nine 1:5 serial dilutions of bispecific antibodies were prepared in triplicate in a deep-well plate (Greiner Bio-One # 786261). 25 µL of diluted bispecific antibody was added to the cells. Expanded T cells were thawed and added at an effector:tumor ratio of 2:1 bringing the volume to 100 µL. The co-culture was incubated at 37 °C with 5% CO_2_ for 48 h. Supernatant was collected and stored at − 80 °C. The level of interferon gamma (IFNγ) was quantified in the supernatants by electrochemiluminescence immunoassay (ECLIA) using the MesoScale Discovery Human IFN-γ Tissue Culture Kit (# K211AEB). The vendor supplied protocol was followed. The plate was read on a MesoScale Discovery Sector Imager 600 instrument. Cytokine concentrations were determined based on a standard curve.

### Proliferation assays

OVCAR3-Luc was harvested and 5 × 10^6^ cells pelleted in a 50 mL conical. 1 mL of Streck Cell Preservative (# 213352) was added and incubated for 30 min at room temperature. The cell pellet was washed twice with PBS, resuspended in T cell media, and counted. 25 × 10^3^ cells in 40 µL was added to a 96-well plate (Corning # 3596). Cells were allowed to adhere overnight at 37 °C with 5% CO_2_. T cells were freshly isolated from frozen PBMCs and labeled with CellTrace Violet Cell Proliferation Kit (invitrogen # C34557) according to vendor specifications. 50 × 10^3^ labeled T cells in 40 µL were added at an effector:tumor ratio of 2:1. Dilutions of bispecific antibodies and washed Dynabeads (gibco # 11161D) were prepared in a deep-well plate (Greiner Bio-One # 786261). A top concentration of 100 nM was 1:5 serially-diluted nine times in T cell media. In a separate well, enough Dynabeads for a ratio of 1 bead: 1 T cell were washed in T cell media according to vendor specifications. Diluted bispecific antibody and washed Dynabeads were stamped onto the 96-well plate at a volume of 40 µL. The co-culture was incubated for 4 days at 37 °C with 5% CO_2_.

T cells were resuspended, transferred to a 96-well U-bottom plate, and pelleted. 25 µL of human Fc Block (BioLegend # 422302) diluted 1:25 in FACS Buffer (autoMACS Running Buffer; Miltenyi Biotec # 130–091-221) was added and incubated for 5 min at room temperature. A master mix of eFluor780 viability dye (diluted 1:1000; eBioscience # 65-0865-14) and 0.5 µL/well of PerCP-Cy5.5 anti-CD3 (BioLegend # 317336), PE-Cy7 anti-CD4 (BioLegend # 344612), and APC CD8a (eBioscience # 17-0087-42) was made. 25 µL of the master mix was added to each well. Antibodies were incubated for 30 min at 4 °C. Cells were washed twice with and resuspended in FACS Buffer. Compensation beads (Invitrogen # 01–2222-42) for eFluor780 and APC were prepared. Data was acquired on the Attune NxT and analyzed using FlowJo software (v10). GraphPad Prism (version 8.1.2) was used to plot the data with a sigmoidal, three parameter, non-linear fit curve.

### CD3 immunohistochemistry

Immunohistochemistry staining for CD3 on select mouse and cynomolgus macaque tissues was performed using formalin fixed paraffin embedded sections. The staining protocol including the deparaffinization and antigen retrieval steps, was performed on a Ventana Discovery XT autostainer using standard Ventana Discovery XT reagents (Ventana, Indianapolis, IN). Slides were deparaffinized then submitted to heat-induced antigen retrieval by covering them with Cell Conditioning 1 (CC1/pH8) solution according to the standard Ventana retrieval protocol. Slides were incubated with the primary rabbit monoclonal antibody 2GV6 (Ventana, Indianapolis, IN) at 0.4 µg/ml or a non-immune isotype-matched control for one hour. Visualization was obtained by incubation with the appropriate Ventana Discovery OmniMap HRP reagent followed by Ventana Discovery ChromoMap 3,3′-Diaminobenzidine (DAB). Counterstaining was performed using Ventana Hematoxylin and Ventana Bluing reagent for 4 min each. Slides were dehydrated, cleared and coverslipped with a synthetic mounting medium.

### In situ hybridization

In situ hybridization was performed on a subset of formalin fixed paraffin embedded tissue sections. In situ hybridization to detect the *Homo sapiens* (Hs) LYPD1, *Mus musculu*s (Mm) LYPD1, *Macaca fascicularis* (Mf) LYPD1, Mf IFNG, Mf IL6, Mf IL1B and Mf TNFA was performed using reagents and equipment supplied by Advanced Cell Diagnostics (ACDBio) (Hayward, CA) and Ventana Medical Systems (Roche, Tuscon AZ). The in situ hybridization BaseScope® probes where designed by ACDBio. Species specific PPIB positive control probe sets and DAPB negative control probe sets were included to ensure mRNA quality and specificity, respectively. The hybridization method followed protocols established by ACDBio and Ventana systems using either 3,3′-Diaminobenzidine (DAB) or Ventana mRNA Red chromogens. Briefly, 5 µm sections were baked at 60 degrees for 60 min and used for hybridization. The deparaffinization and rehydration protocol was performed using a Sakura Tissue-Tek DR5 stainer with the following steps: 3 times xylene for 5 min each; 2 times 100% alcohol for 2 min; air dried for 5 min. Off-line manual pretreatment used 1X retrieval buffer at 98 to 104 degrees C for 10 min. Following pretreatment the slides were transferred to a Ventana Ultra autostainer to complete the ISH procedure including protease pretreatment; hybridization at 43 degrees C for 2 h followed by amplification; and detection with HRP and hematoxylin counter stain. Optimization was performed by first evaluating PPIB and DAPB hybridization signal and subsequently using the same conditions for all slides.

### Collection of patient specimens

All human primary tumor specimens, which were used for limited tumor epidemiology, were collected according to Institutional Review Board protocol PG-ONC-2003/2 approved by the ethics committee N.N. BLOKHIN NMRCO MHRF/Russian Oncological Research Center, and informed consent was confirmed by the Novartis Institutes for BioMedical Research Human Tissue Network. Since Novartis commercially sourced these human tissue samples from N.N. BlOKHIN NMRCO MHRF/Russian Oncological Research Center, none of the authors affiliated with this paper are from this N.N. BLOKHIN NMRCO MHRF. When sourcing human tumor and normal tissue samples, Novartis adheres to strict guidelines, including obtaining and using only fully consented samples under IRB approval. All methods were performed in accordance with the relevant guidelines and regulations.

### Animal care and use statement

All animal studies were conducted in compliance with the Animal Welfare Act, the Guide for the Care and Use of Laboratory Animals and the Office of Laboratory Animal Welfare. All procedures were approved by the Novartis Institutes for BioMedical Research Institutional Animal Care and Use Committee (IACUC) and were conducted in accordance with the guidelines as published in the Guide for the Care and Use of Laboratory Animals. This study is reported in accordance with ARRIVE guidelines.

## Supplementary Information

Supplementary Tables.

Supplementary Information.
