# Supplementary material for: PAX8 lineage-driven T cell engaging antibody for the treatment of high-grade serous ovarian cancer
Source: Sci Rep. 2021 Jul 21;11:14841. doi: 10.1038/s41598-021-93992-1 (PMC8295318; doi:10.1038/s41598-021-93992-1)
Supplement: Supplementary file 2 — Supplementary Information. [file 41598_2021_93992_MOESM2_ESM.pdf]

PAX8 lineage-driven T-cell engaging antibody for the treatment of high-grade serous ovarian cancer

Emily Lee[1], Sarah Szvetecz[1], Ryan Polli[3], Angelo Grauel[4], Jayson Chen[5], Joyce Judge[6], Smita Jaiswal[6], Rie Maeda[1], Stephanie Schwartz[4], Bernd Voedisch[7], Mateusz Piksa[7], Chietara Japutra[2], Lingheswar Sadhasivam[2], Yiqin Wang[2], Ana Carrion[2], Sinan Isim[1], Jinsheng Liang[1], Thomas Nicholson[1], Hong Lei[1], Qing Fang[1], Michelle Steinkrauss[6], Dana Walker[6], Joel Wagner[1], Viviana Cremasco[4], Hui Qin Wang[1], Giorgio G Galli[8], Brian Granda[2], Keith Mansfield[6], Quincey Simmons[6], Andrew Anh Nguyen[2*], Nicole Vincent Jordan[1*]

Supplementary Methods:

scRNA-seq analysis. Single-cell RNA-seq data from the Gene Expression Omnibus (GSE146026) (1)was analyzed. The portion of data from the 10X Genomics platform was used for the main analysis (GSE146026_Izar_HGSOC_ascites_10x_log.tsv.gz), comprised of 8 HGSOC samples and 9,609 cells. Because the cell type labels were not provided in the supplement, cell type labels were rederived. The cells were clustered using the FindClusters function in Seurat v3.0 (2) and visualized using the RunTSNE function, using default parameters in both cases. We annotated the clusters using cell type markers from Izar et al., macrophages (*CD14, CD68*), fibroblasts (*PDPN, COL1A1, COL1A2*), epithelial cells (*EPCAM*), dendritic cells (*CD83, CCR7*), natural killer cells (*NKG7*), and T cells (CD3D/E/G). For calculating the fraction of cells expressing a gene, any cells with non-zero expression of that gene were considered to be expressing that gene. The smart-seq2 data (GSE146026_Izar_HGSOC_ascites_SS2_log.tsv.gz) was clustered using the same methods as described above and then used to visualize the co-expression of LYPD1 and PAX8, in addition to the 10x data. This visualization was done using the Visualization in Plugin tool (Li et al., bioRxiv, https://doi.org/10.1101/2020.08.28.270652) Dual Genes function in cellxgene (https://github.com/chanzuckerberg/cellxgene).

ChIP-seq and analysis. ChIP-seq was performed as previously described (3). Cells were cross-linked in 1% formaldehyde for 10 min at room temperature. The reaction was stopped by addition of 0.125M

glycine, and dells were lysed and harvested in ChIP buffer (100 mM Tris at pH 8.6, 0.3% SDS, 1.7% Triton X-100, and 5 mM EDTA). The chromatin disrupted by sonication using a EpiShear sonicator (Active Motif) to obtain fragments of average 200–500 bp in size. Suitable amounts of chromatin were incubated with specific antibodies overnight. Antibodies used are: PAX8 (1.5ug in 800 ul, Cell Signaling, 59019). Immunoprecipitated complexes were recovered on Protein G Dynabeads (Invitrogen), and DNA was recovered by reverse crosslinking and purified using SPRI Select beads (Beckman Coulter). Libraries for ChIP-sequencing were generated using Ovation® Ultralow Library System V2 (NuGEN), and barcodes were added using NEBNext Multiplex Oligos for Illumina (Index Primers Set 1) (NEB) according to the manufacturer's recommendation.

Bispecific antibodies. *Cloning, expression, and purification*

Amino acid sequences for anti-CD3 and anti-LYPD1 obtained from previous publications or patents, and listed. All gene cassettes of the heterodimeric bispecific antibodies were synthesized by GeneArt (ThermoFisher Scientific), cloned into our proprietary vectors and designed to have knobs into holes mutations (4) and DANAPA IgG1 silencing in the Fc module. The anti-CD3 arm of the 1+1, tandem fab, and cis 2+1 bispecific format **(Figure 3A)** consisted of the plasmid encoding the anti-CD3 scFv-hinge-C_H2-C_H3 (knob mutations) polypeptide. The anti-CD3 arm of the 2+1 bispecific format is identical as described above except this bicistronic plasmid also encodes the anti-LYPD1 light chain polypeptide and the anti-LYPD1 V_H/CH1 fused to the N-terminus of the anti-CD3 polypeptide separated by $(G_4S)_2$ linker.

For the anti-LYPD1 arm used in the 1+1 and 2+1 bispecific formats, the bicistronic plasmids encodes the anti-LYPD1 kappa light chain polypeptide and heavy chain (hole mutations) polypeptide. For the tandem anti-LYPD1 fab format, this plasmid is similar to the previous anti-LYPD1 construct except its heavy chain having the $(G_4S)_2$ linker fused to the tandem repeats of V_H/CH1. The anti-LYPD1 plasmid used in the cis 2+1 format is similar to the 1+1 anti-LYPD1 construct except also having anti-LYPD1 V_H/CH1 fused to the C-terminus of Fc (Proline^{449}) separated by $(G_4S)_4$ linker.

All plasmids were sequence verified and transformed into *E.coli* for scale-up DNA extraction. All proteins were transiently expressed in Expi293F cells using polyethylenimine hydrochloride (PEI-MAX, linear MW 40K; Polysciences) in 1:3 DNA to PEI-MAX ratio. DNA ratio used was 1:1 for anti-CD3 to anti-LYPD1

plasmid. After 5-7 days of expression, the clarified cell culture supernatant was purified on an ÄKTA FPLC instrument by affinity purification using MabSelect Sure column from Cytiva LifeSciences followed by CaptureSelect™ CH1-XL column from Thermo Fisher Scientific to obtain heterodimeric bispecifics as per manufacturer's protocols. Proteins used in nonhuman primate study were also passed on Cytiva's Superdex 200 gel filtration column. Prior to use, the purity of the bispecific antibodies was confirmed by SDS-PAGE, aggregation by analytical size exclusion chromatography, and correct heterodimeric formation by mass spectrometry.

16nM anti-CD3 SP34 scFv	EVQLVESGGGLVQPGGSLKLSCAASGFTFNTYAMNWVRQASGKGLEWVGRIRSK<br>YNNYATYYADSVKDRFTISRDDSKSTLYLQMNSLKTEDTAVYYCVRHGNFGNSYVS<br>WFAYWGQGTLVTVSSGGGGSGGGGSGGGGSGGGGSQAVVTQEPSLTVSPGGT<br>VTLTCRSSTGAVTTSNYANWVQQKPGQAPRGLIGGTNKRAPWTPARFSGSLLGDK<br>AALTLSGAQPEDEAEYFCALWYSNLWVFGGGTKLTVLG
5nM anti-CD3 H2C scFv	EVQLVESGGGLVQPGGSLKLSCAASGFTFNKYAMNWVRQAPGKGLEWVARIRSK<br>YNNYATYYADSVKDRFTISRDDSKNTAYLQMNNLKTEDTAVYYCVRHGNFGNSYIS<br>YWAYWGQGTLVTVSSGGGGSGGGGSGGGGSGGGGSQTVVTQEPSLTVSPGGT<br>VTLTCGSSTGAVTSGNYPNWVQQKPGQAPRGLIGGTKFLAPGTPARFSGSLLGGK<br>AALTLSGVQPEDEAEYYCVLWYSNRWVFGGGTKLTVLG
Low affinity anti-LYPD1 V_H	EVQLVESGGGLVQPGGSLRLSCAASGFTITNYGIHWVRQAPGKGLEWVGRIYPDS<br>GATYYADSVKGRFTISADTSKNTAYLQMNSLRAEDTAVYYCARKLWVSRAGMDYW<br>GQGTLVTVSS
Low affinity anti-LYPD1 V_L	DIQMTQSPSSLSASVGDRVTITCRASQDVSTAVAWYQQKPGKAPKLLIYSASFLYS<br>GVPSRFSGSGSGTDFTLTISSLQPEDFATYYCQQSYTTPPTFGQGTKVEIK
High affinity anti-LYPD1 V_H	EVQLVESGGGLVQPGGSLRLSCAASGFTITNYGIHWVRQAPGKGLEWVGRIYPDS<br>GATYYADSVKGRFTISAD TSKNTAYLQMNSLRAEDTAVYYCARKLWISIAGMDYW<br>GQGTLVTVSS
High affinity anti-LYPD1 V_L	DIQMTQSPSSLSASVGDRVTITCRASQDVSTAVAWYQQKPGKAPKLLIYSASFLYS<br>GVPSRFSGSGSGTDFTLTISSLQPEDFATYYCQHSYATPFTFGQGTKVEIK

Cell line models. OVCAR3 (ATCC, USA; # HTB-161) is a patient-derived ovarian adenocarcinoma cell line; Kuramochi (Japanese Collection of Research Bioresources Cell Bank, Japan; # JCRB0098) is a patient-derived ovarian serous adenocarcinoma; OVSAHO is a patient-derived ovarian carcinoma (Japanese Collection of Research Bioresources Cell Bank, Japan; # JCRB1046); JHOS-2 (Riken, Japan; # RCB1521) is a patient-derived ovarian serous adenocarcinoma; DMS273 (European Collection of Authenticated Cell Cultures, England; # 95062830) is a patient-derived small cell lung carcinoma cell line;

U-251 MG (Health Science Research Resources Bank, Japan; # IFO50288) is a patient-derived glioblastoma astrocytoma cell line; and OVCAR8 (National Cancer Institute Division of Cancer Treatment & Diagnosis, USA) is a patient-derived high grade ovarian serous adenocarcinoma cell line. DMS273-LYPD1-OE was engineered by lentiviral infection with a plasmid prepared by Gateway cloning (Life tech # 11791100) LYPD1 plasmid into the pEF1a-V5-His vector (Novartis). All cell lines were luciferized with an EF1a-Luciferase (firefly) virus (GenTarget # LVP435) following vendor specifications.

Tissue digestion. Tissues were digested using the method described in Cremasco 2018 and Grauel 2020. Briefly, tissues were minced into fine pieces (approximately 1 mm^3), transferred into 15 mL conical tubes containing 2 mL of digestion buffer [RPMI (Gibco), 2% FBS, 0.2 mg/mL Collagenase P (Roche), 0.2 mg/mL Dispase (Gibco), and 0.1 mg/mL DNase I (Roche)], and placed into a water bath at 37°C. Tissue fragments were subjected to consecutive cycles of agitation/pipetting, and the supernatant containing freed cells was collected every 20 minutes and quenched at 4°C in 50 mL conical tubes containing cold flow cytometry buffer (PBS, 2% FBS, and 2 mmol/L-EDTA). When the digestion was completed, cells were filtered through a 70-µm mesh, centrifuged and used for subsequent analyses.

Biacore K_D Determination. The affinity and avidity effect of anti-LYPD1 bispecific antibodies binding with human LYPD1 were assessed via SPR using the Biacore T200 instrument (Cytiva, formerly GE Healthcare Lifesciences) at 25°C as described below. The Biotin Capture kit (Cytiva, Cat# 28920234) was utilized, and HBS-EP+ (Cytiva, Cat# BR100669) with addition of NSB reducer (Cytiva, Cat#BR100691) was used as running buffer and sample dilution buffer in this experiment.

After running the biotin capture solution over all 4 flow cells at 2ul/min for 60sec, the biotinylated human LYPD1 protein was injected at a flow rate of 10ul/min sequentially over Fc (flow cell) 2, Fc3, and Fc4 to get approximately 170RU, 75RU and 50RU capture levels respectively. Antibody samples with 1:2 serial dilution ranging from 1.56nM to 200nM were then injected at 60ul/min for 240sec. Dissociation was followed for 800sec. Regeneration was done at end of each cycle with injection of supplied regeneration buffer at 20ul/min for 60sec. Double reference subtraction was completed to generate the final data. Fitting was performed using a 1:1 binding model (parameter Rmax fitted locally; RI fitted locally).

Antibody binding capacity determination. Tumor cell lines were harvested with Accutase (Sigma # A6964) and added to a 96-well U-bottom plate (Corning # 3799) at a density of 0.1 × 10^6 cells per well. Cell pellets were resuspended in 30 nM of UKP391 antibody and isotype control (diluted in FACS Buffer). Cells were incubated for 1 hour at 4℃. In parallel, Quantum Simply Cellular anti-Human IgG beads (Bangs Laboratories # 816) were prepared per vendor specifications.

Generation of CD3ε Chimeric Knockin Mice. The chimeric CD3ε knockin (KI) mouse were derived internally at Novartis Institutes for BioMedical Research Institutional (Cambridge, MA). In brief, four guide RNAs (gRNAs) and a plasmid vector (6.2 Kb) containing human protein sequence (DGNEEMGGITQTPYKVSISGTSVELT) were designed to target the mouse *CD3ε* allele. These gRNAs, # 4-1 (forward sequence: 5' ctttcagatgccgagaacat) , #4-2 (complementary sequence: 5'-aatcttcttaccaatgttct), #5-2 (complementary sequence: 5'-aagttctcgtcactgtctag), #5-3 (complementary sequence: 5'-agggcacgtcaactctacac) were ordered as crRNAs from IDT as part of their Alt-R CRISPR/Cas9 system. A pair of crRNAs (4-1/5-3 or 4-2/5-2) were microinjected, along with the universal Alt-R tracrRNA (IDT), plasmid vector and Cas9 protein (PNA Bio), into the pronucleus of fertilized C57Bl/6J oocytes, viable 2-cell embryos were re-implanted in pseudo-pregnant B6CBAF1 female mice. The resulting pups were genotyped by PCR, SacI Digestion and Sanger sequencing. The founders were backcrossed with C57BL/6J (JAX, 000664). Three lines, II544, II558, II572 were confirmed by Targeted Locus Amplification technology (Cergentis, Utrecht). For final genotyping, Primer: 5F3: 5'-CCTGGTGCATAGCCAACAGATATAG, 5R3: 5'-CTTCCACACCTACTCATTGTGTGCAG for wildtype, 5R4: 5'-CTTCTTACGTGTCTGTGTAATACCACCC for the human sequence. PCR condition: denature at 95°C for 20 sec, annealing at 60°C for 20 sec, elongation at 72°C for 45 sec, for 35 cycles. These primers are predicted to give a band of 618 base pairs for wild type mouse, and 228 base pairs for heterozygous mouse.

The chimeric CD3ε KI mouse is able to bind to N-terminal SP34 epitope of anti-CD3 antibody which enabled us to test the bispecific CD3 antibody for on target off tissue toxicity and therapeutic efficacy in this mouse model. The function of chimeric CD3ε was confirmed by using CD20-CD3 bispecific antibody to reduce B cell number in PBMCs in the transgenic mice.

Cytokine release and histology analysis. The female homozygous chimeric CD3ε KI mouse at 12 weeks of age were administered with control IgG (3 mg/kg), VHP354 (0.1 or 1.0 mg/kg) or QZC131 (0.3 or 3 mg/kg) by intravenous injection. Blood was collected at 6 hrs and 24 hrs post dose for cytokine analysis. On day 7, mice were euthanized, and whole body was unzipped, pituitary was exposed and the whole mouse was fixed in 10% formalin for 24hrs and then transferred to 70% ethanol.

U251MG xenograft adoptive transfer model. All animal studies were approved by the Novartis Institutes for BioMedical Research Institutional Animal Care and Use Committee (IACUC) and were conducted in accordance with the guidelines published in the Guide for the Care and Use of Laboratory Animals. All animals had access to food and water ad libitum. Eight weeks old female NSG mice (The Jackson Laboratory, Bar Harbor, ME) were implanted subcutaneously on the right flank with 5×10^6 U251MG cells on Day 0, and frozen human PBMC at 10 $\times10^6$ was administered on Day 22 by intravenous injection. When tumor volume reached an average of 200-300 mm3 on Day 34, the animals were randomized and LYPD1 bispecific antibodies (VHP354, QWG430 or QZC131) or control antibody were given by intravenous injection on Day 35. Tumor volume and body weight were measured twice per week until the end of the study.

NHP toxicology study design. Non-naive Cynomolgus monkeys (*Macaca fascicularis*) were administered QZC131 or VHP354, once via intravenous injection according to the study design **(SupplementaryTable 1)**.

The in-life portion was performed by Covance in Madison, WI.

Data for Anatomic Pathology was captured using the departmental computerized systems (Pristima™ software, version 6.3.2).

Study design, animal allocation and test article doses

Group	Treatment	Dose Level (mg/kg/day)	Animal number
			Males
1	QZC131 (CD3-LYPD1, low affinity)	0.3	P0001
2	QZC131 (CD3-LYPD1, low affinity)	3.0	P0101-0102
3	VHP354 (CD3-LPD1, high affinity)	0.1	P0201
4	QZC131 (CD3-LYPD1, low affinity)	1.0	P0301-0302

Anatomic pathology sampling and processing. At the completion of the study (Study Day 8), approximately 7 days hours after the single dose, animals per group were submitted for necropsy. All animals submitted for necropsy had brain, kidney (paired) and pituitary dissected and weighed. Absolute and relative organ weights were calculated. The brain, pituitary gland, pancreas, liver, colon, kidneys (2), Animal ID, injection site and any lesions were collected and processed for histopathologic examination.

Toxicokinetics Study, Sample, and Data Analysis. The purpose of this study was to generate primate toxicokinetic data and determine toxicity of anti-LYPD1 T-cell dependent bispecific antibodies (TDB), QZC131 and VHP354. Cynomolgus monkeys aged 4 to 7 years, weighing 5 to 11 kg, were provided by Covance Laboratories, Inc. Animals were administered intravenously 0.3, 1, 3 mg/kg QZC131, or 0.1 mg/kg VHP354 via saphenous vein in a total volume equal to 1 mL/kg and observed for 7 days. During this period, approximately 1 mL blood samples were harvested at pre-dose, 0.5, 1, 4, 12, 24, 72, 96, and 168 h post-dose from the femoral vein. Blood samples were held at room temperature for 30 min and allowed to clot prior to centrifugation. Resulting serum was aliquoted and stored at -60 to -80°C until shipment.

Bioanalytical assessment of cynomologus serum was conducted by BioAgilytixs (Durham, NC) using ECLIA. Briefly, streptavidin gold MSD plate wells were blocked with 150 µL/well of SuperBlock Blocking Buffer in PBS and incubated at room temperature for 1 h with shaking at 600 rpm. Following the blocking step, the plate was washed three times with 300 µL/well of 1X PBS + 0.05% Tween 20. The plate was coated with 50 µL/well of 0.2 µg/mL biotinylated goat anti-human IgG in PBS and incubated at room temperature for 1h with shaking at 600 rpm. During this incubation, cynomolgus monkey serum was centrifuged at 2,000 rpm for 10 minutes where then standards, controls, and samples were prepared and or diluted to the minimum required dilution (1:50). Following these steps, the plate was washed three times with 300 µL/well of 1X PBS + 0.05% Tween20. Standards, assay controls, and samples were added to the plate at a volume of 50 µL/well in duplicate and incubated at room temperature for 2 h with shaking at 600 rpm. After the sample incubation, the plate was washed three times with 300 µL/well of 1X PBS + 0.05% Tween 20. The detection antibody, 50 µL/well of 0.1 µg/mL Ru-Goat anti-human IgG in assay buffer, was added to the plate and incubated at room temperature for 2 h with shaking at 600 rpm. After incubation, the plate was washed three times with 300 µL/well of 1X PBS + 0.05% Tween 20 and

tapped dry on absorbent paper. MSD Read Buffer Gold was added to each well of the plate at 150 µL. The plate was covered and incubated for 6-8 minutes before being read on the MSD Sector Imager 600 plate reader.

A non-compartmental analysis of the pharmacokinetic data obtained from BioAgilytix was conducted using Phoenix WinNonlin®. AUC was estimated using linear up log down method and CL was calculated as Dose / AUC_{inf}. Other PK parameters (i.e C_{max}, $t_{1/2,terminal}$, & V_{SS}) were taken directly from the NCA output.

Immunoassay measurements and analyses from NHP serum. Serum samples received from the testing facility were stored at ≤ -70°C until analysis. These samples represented blood collections at dosing phase days 1 at predose and approximately 6 and 24 hours postdose. Samples were tested with the kit assay, Cytokine Monkey Magnetic 29-Plex Panel (Invitrogen, cat. No. LPC0005M, Lot # 1833398) using Luminex xMAP technology platform. Testing procedure conformed to Novartis Working Instructions for the Multiplex Luminex® Method for Quantitative Detection of Monkey Cytokines. Analysis was based on the fluorescence intensity minus background (FI-Bkgd) extrapolation from a five parameter (5-PL) curve fitting program as a part of the Bio-Plex manager Software 6.1 analysis platform as a part of the Bio-Plex-200. Values were graphed (for report illustrative purposes only) in GraphPad Prism v7.02 (GraphPad Software, Inc., La Jolla California USA, www.graphpad.com). The specific analytes assessed were IL-1β, IL-1RA, IL-2, IL-5, IL-6, IL-10, IL-12p70, IL-17, IFN-γ, IP-10, MCP-1 and TNFα. Changes 2-fold or greater of predose value were considered notable.

Calculations. Cytotoxicity was calculated as a percentage of the untreated wells using the following calculation:

(1 – (raw luminescence of treated / average raw luminescence of untreated)) x 100%

The normalized values for each condition were averaged and plotted in GraphPad Prism (version 8.1.2) against the respective concentrations. A sigmoidal, three parameter, non-linear regression analysis was used to determine the IC50.

Similarly, FACS EC50s were determined by plotting geometric mean fluorescence intensity values, as calculated by FlowJo software (v10), against the respective concentrations and plotted with a sigmoidal, three parameter, non-linear regression analysis in GraphPad.

Fold change for EC50 and A_{max} were calculated using the following equations:

Fold change = $A_{max, timepoint}/A_{max, predose}$

Fold change = $EC50_{timepoint}/EC50_{predose}$

Fold differential values for all other assays were calculated using the following equation:

Fold difference = $Value_{Bispecific}/Value_{isotype\ control}$

References

1. Izar B, Tirosh I, Stover EH, Wakiro I, Cuoco MS and Regev A et al. A single-cell landscape of high-grade serous ovarian cancer. Nature Med. **26**:1271-1279 (2020).
2. Stuart T, Butler A, Hoffman P, Hafemeister C, Papalexi E, III WMM, Hao Y, Stoeckius M, Smibert P, Satija R. Comprehensive integration of single-cell data. Cell. 177: 1888-1902 (2019).
3. Bleu M, Swann G, Lopes R, Sprouffske K, Apfel V and Galli GG et al. PAX8 activates metabolic genes via enhancer elements in renal cell carcinoma. Nat Comm. **10**(3739):1-10 (2019).
4. Merchant AM, Zhu Z, Yuan JQ, Goddard A, Adams CW, Presta LG, et al. An efficient route to human bispecific IgG. Nat Biotechnol. (1998) 16:677–81.

Supplementary Figure 1

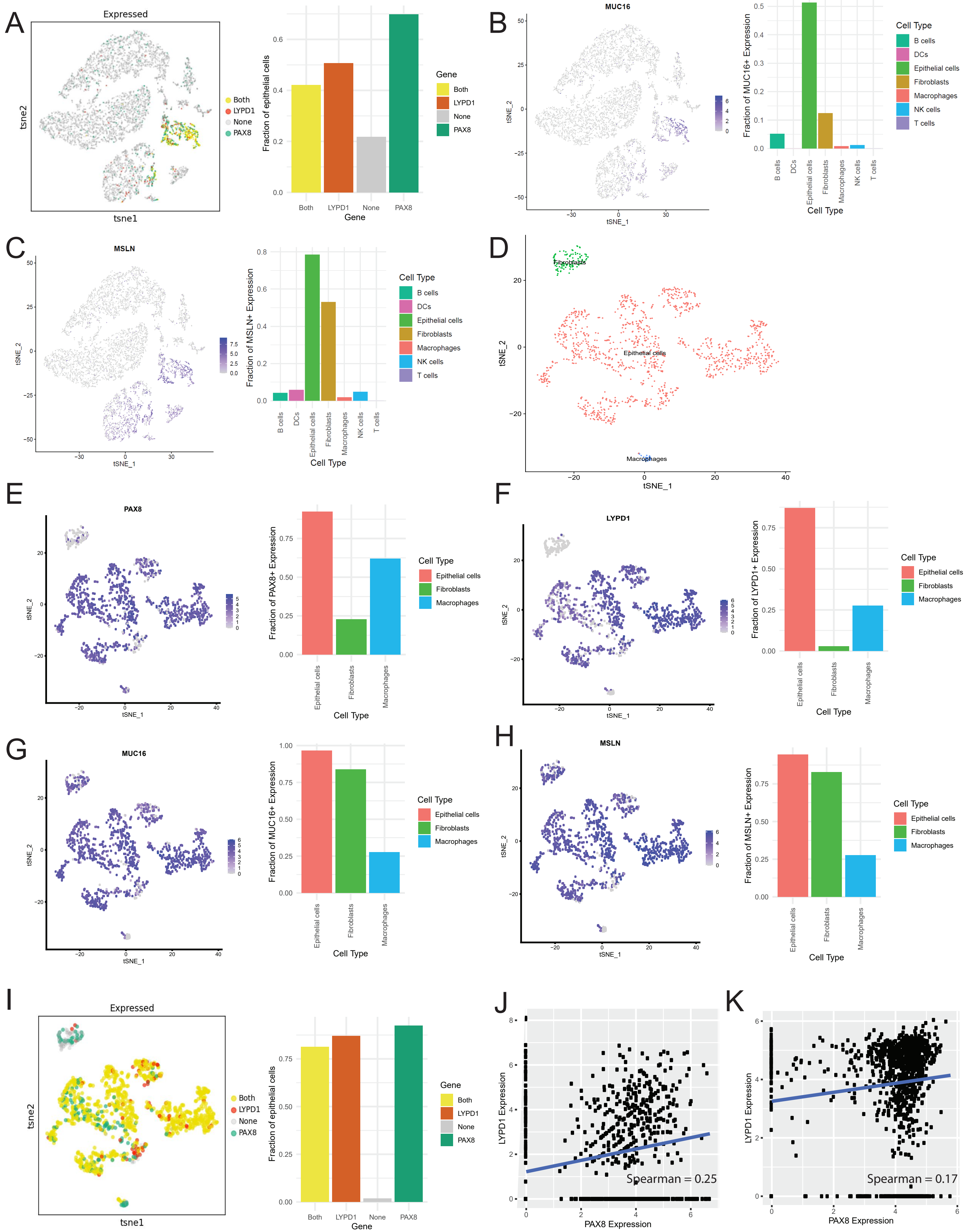

Supplementary Figure 2

A

score	0	1	2	3	4

		LYPD1			Human-LYPD1	
Tissue	Human	Cyno	Mouse	Mouse-NSG	GTEx	
Brain-cerebellar cortex (neurons)	3	4	ND	4	9.223	Neurons positive
Pituitary	3	2	1	3	25.32	Anterior pituitary
Colon	1	2	ND	2	3.894	Predominantly in epithelial cells
Fallopian tube	4	3	2	2	2.388	
Pancreas (ducts)	1	1	ND	1	0.984	supporting cells in ducts are positive
Liver (periductular cells)	ND	2	ND	2	0.114	supporting cells in ducts are positive
Peripheral Nerve	1	1	ND	1	1.715	

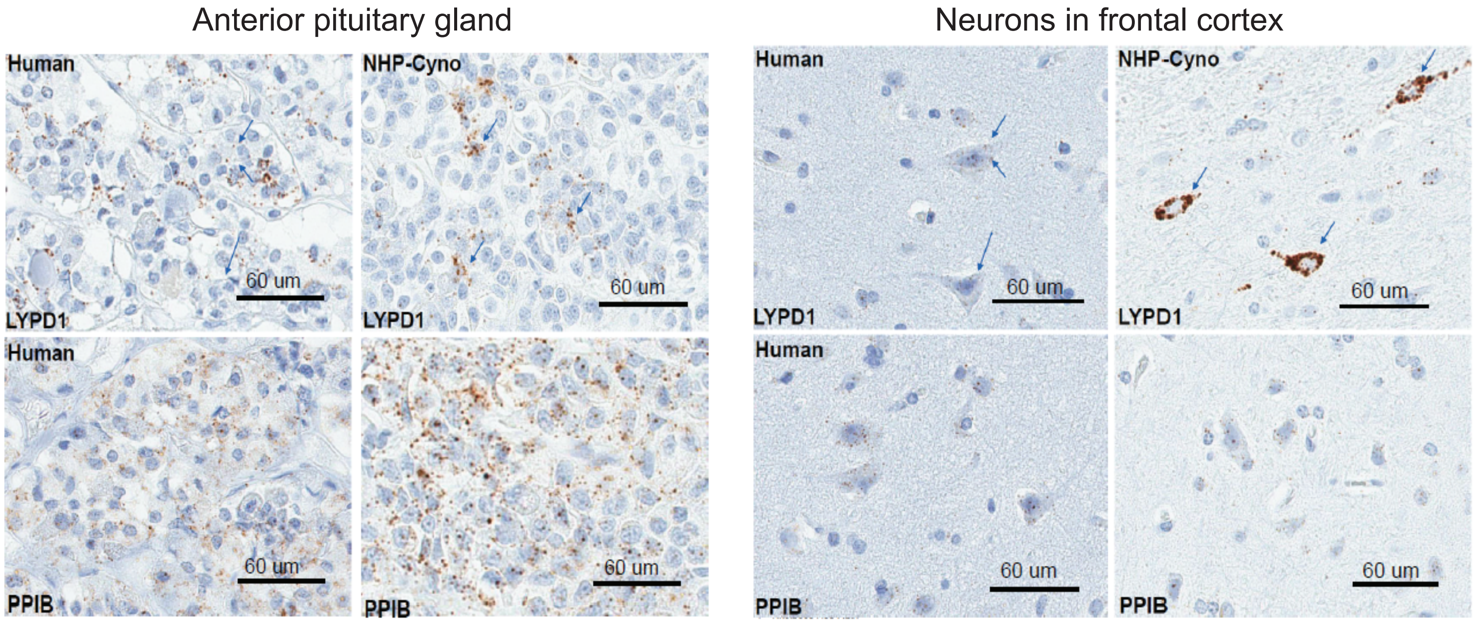

B

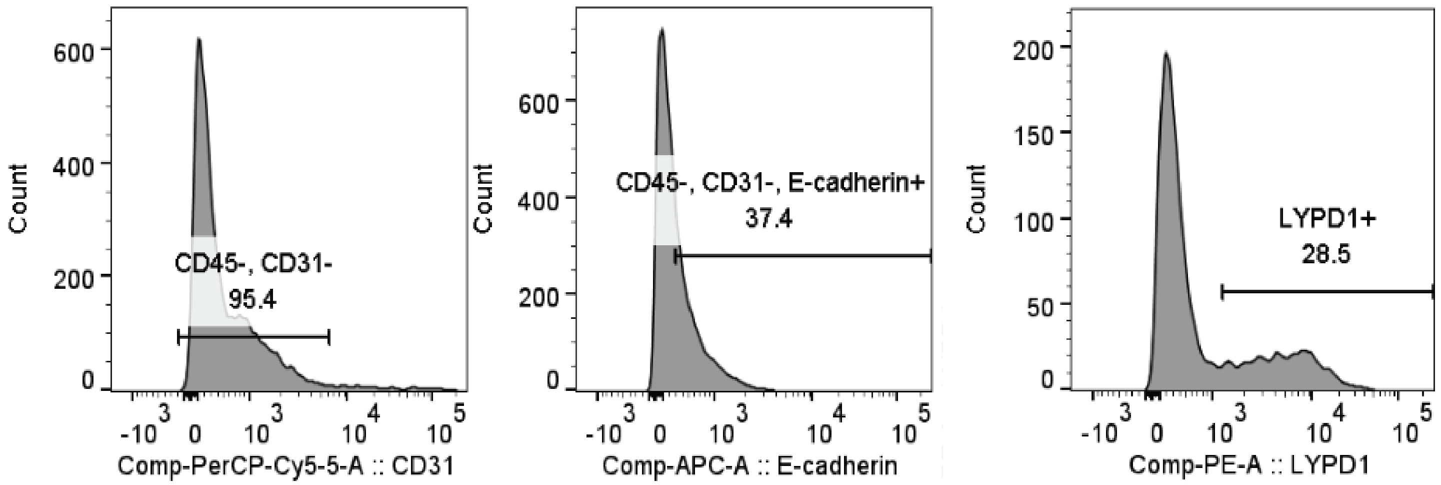

Supplementary Figure 3

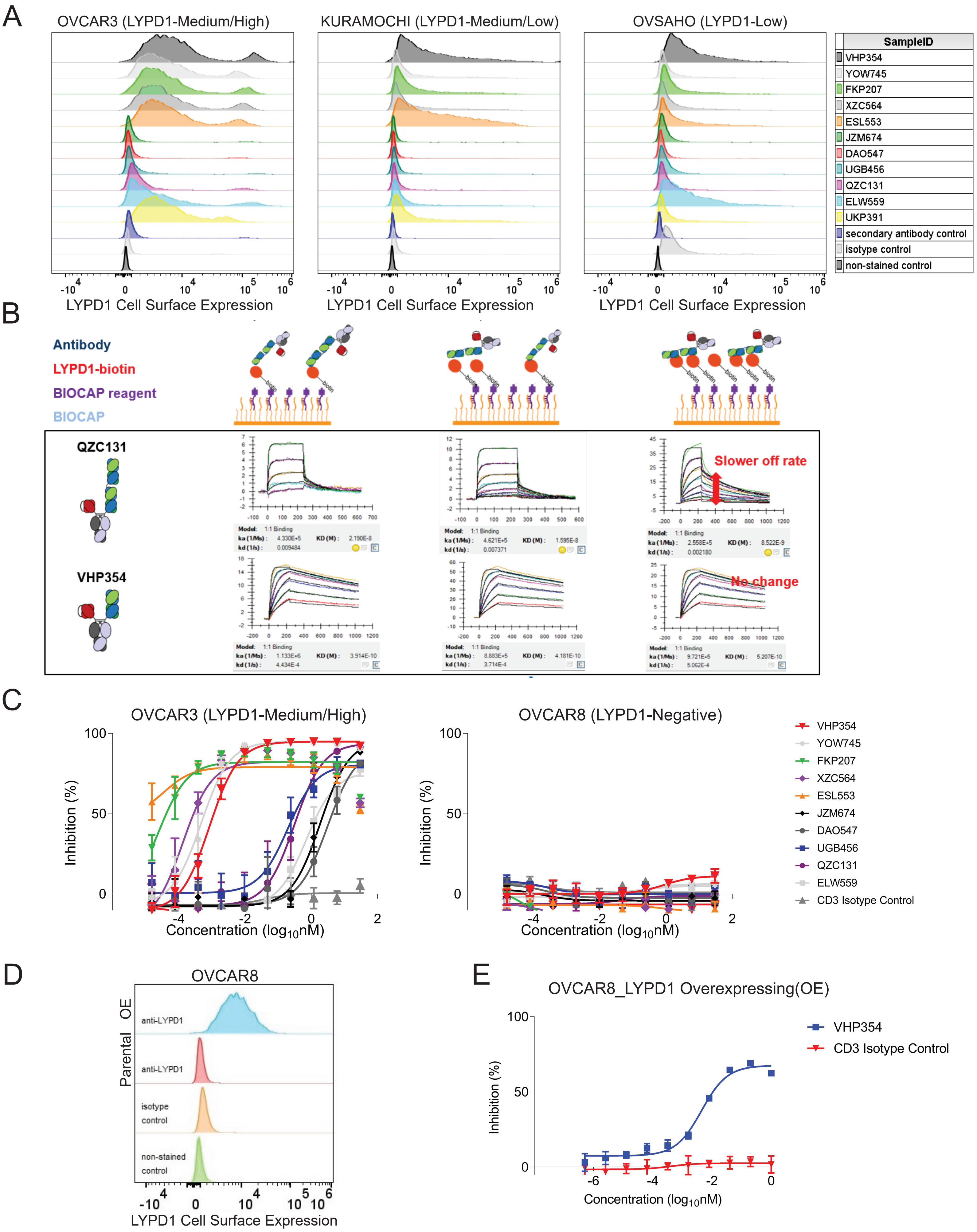

Supplementary Figure 4

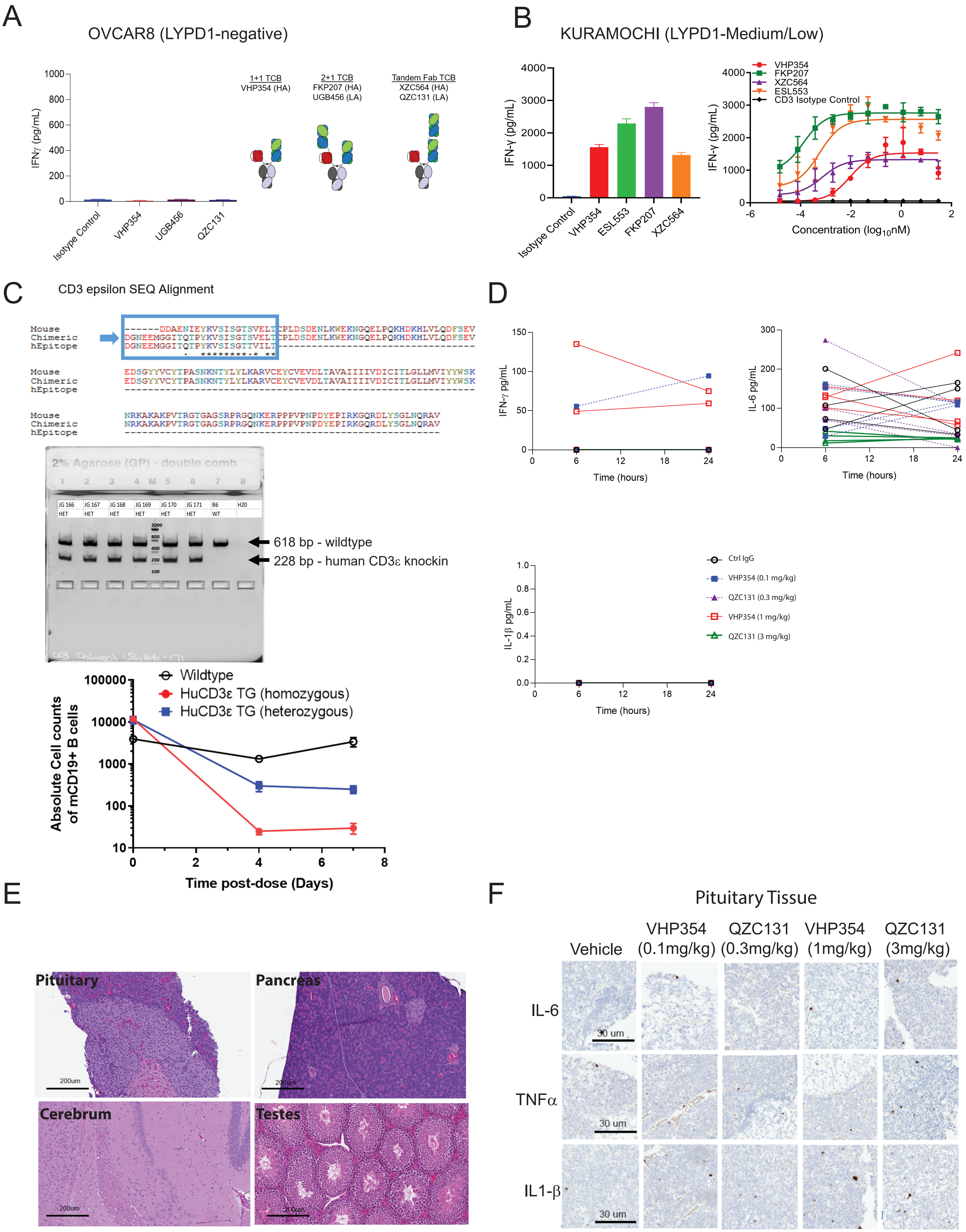

Supplementary Figure 5

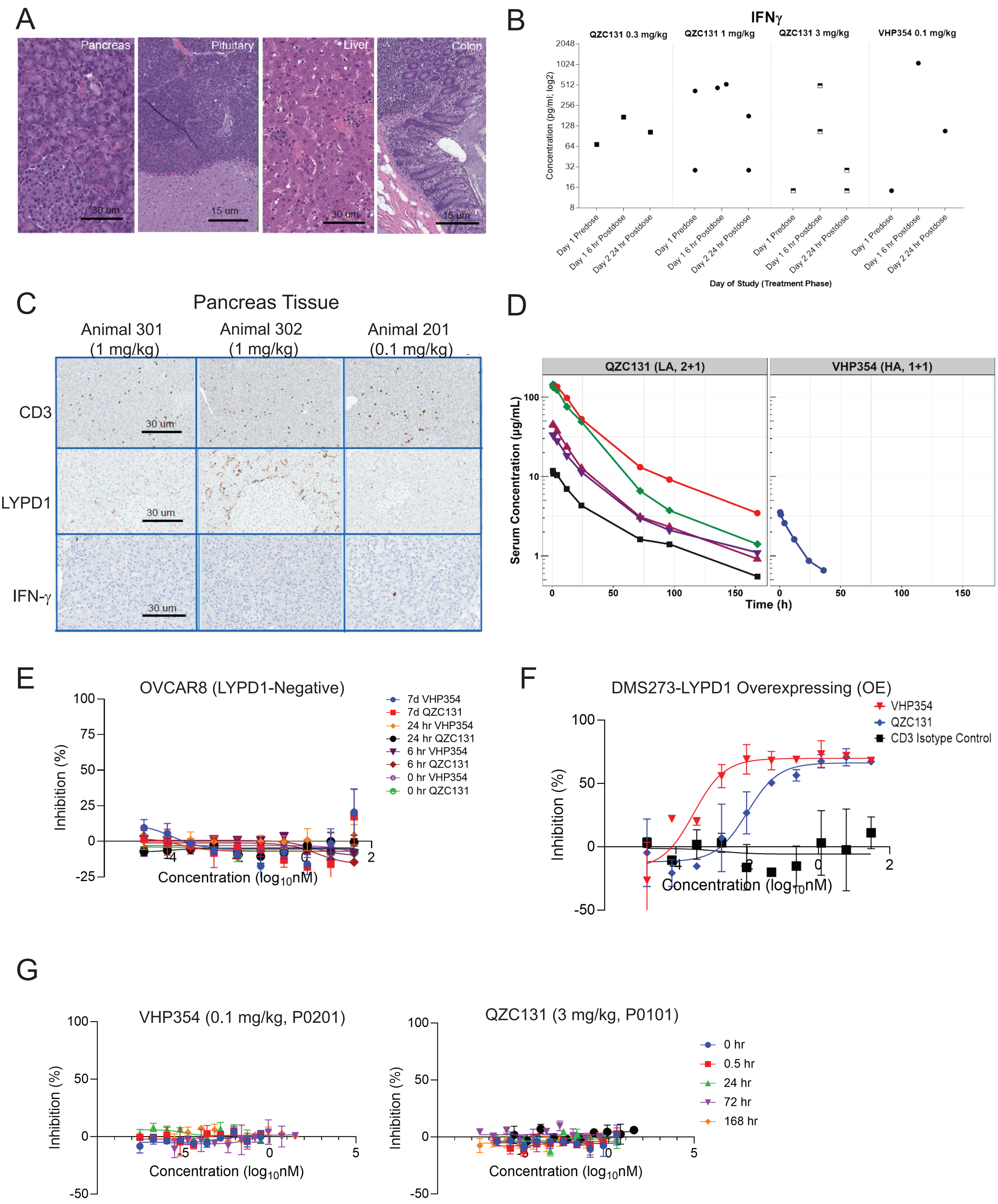

Supplementary Figure 6

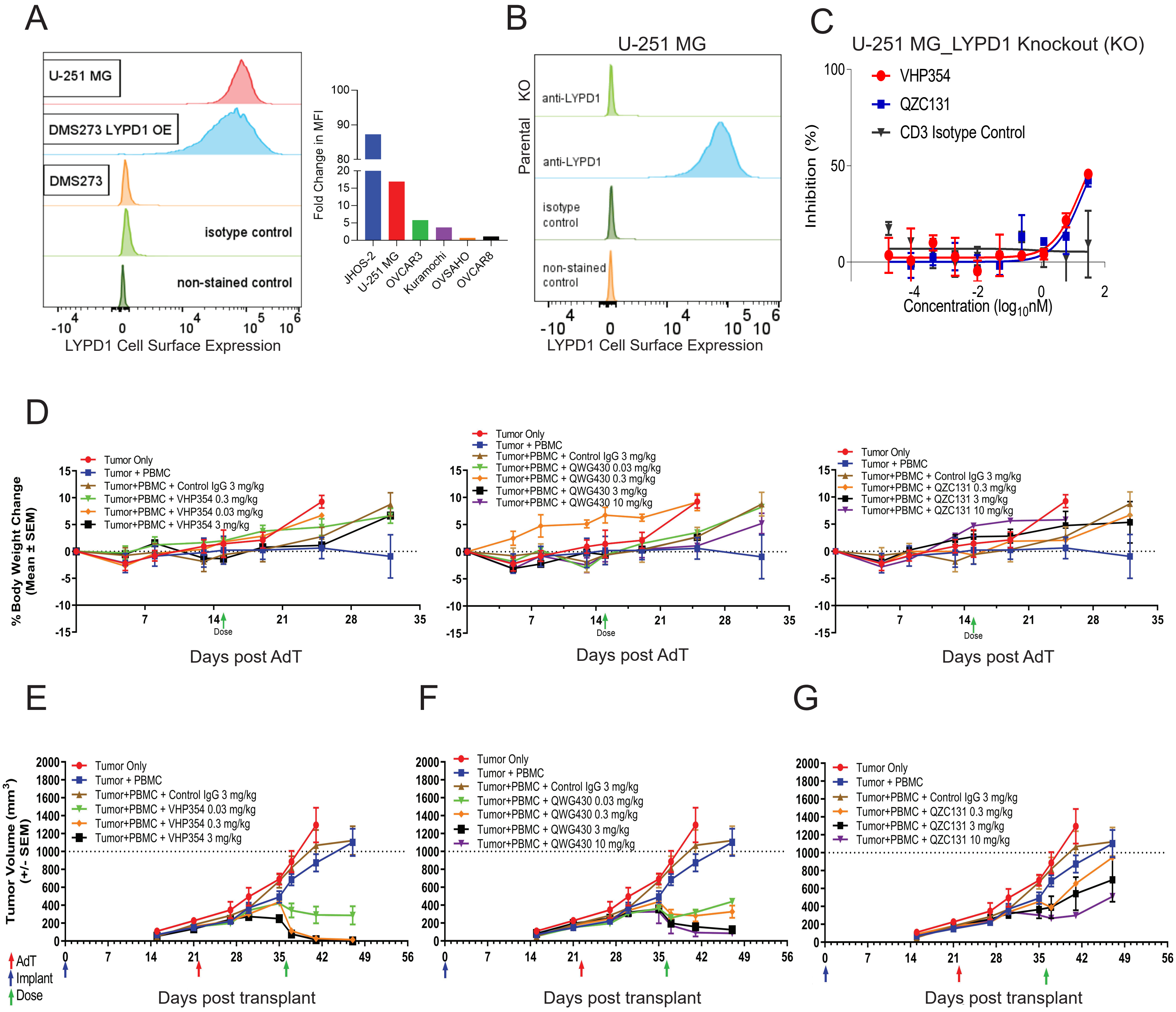

Supplementary Figure Legends:

Supplementary Figure 1: LYPD1 is a PAX8 lineage-driven target with expression limited to malignant epithelial tissues. (a) TSNE plot of scRNA-seq 10X demonstrates epithelial compartment co-expression of LYPD1 and PAX8 (yellow), LYPD1 (red) or PAX8 (green) in 22 ascites samples across 11 patients. (b-c) TSNE plot of 35,947 single cells derived from the ascites of 11 HGSOC patients shows ovarian cancer targets MUC16 and MSLN expressed in both the epithelial tumor cell and fibroblast populations at 51.3% and 12.4% frequency for MUC16 and 78.5% and 53.1% frequency for MSLN. (d) TSNE plot of scRNA-seq SMART-seq2 of 1297 sorted EPCAM+/CD24+ epithelial cells demonstrates malignant (epithelial) and non-malignant (fibroblast and macrophages). (e-h) epithelial (red) compartment expression in >80% of cells for PAX8 (e), LYPD1 (f), MUC16 (g) and MSLN (h) with only MUC16 and MSLN having expression in >80% of fibroblasts (green). (i) TSNE plot of scRNA-seq SMART-seq2 of 1297 sorted EPCAM+/CD24+ epithelial cells demonstrates co-expression of LYPD1 and PAX8 (yellow) in 81.3% of cells sequenced. (j-k) Spearman correlation between LYPD1 and PAX8 for scRNA-seq 10X (j) and SMART-seq (k), respectively.

Supplementary Figure 2: LYPD1 normal expression is limited to lineage tissues.

(a) Representative RNA-ISH demonstrates moderate levels of LYPD1 RNA expression distributed across the HGSOC tissues of origin, the anterior pituitary and neurons in the frontal cortex with limited expression across other critical normal tissues. Scale bar marks 60 um. (b) FACS histograms show LYPD1 expression in 28.5% of dissociated pituitary cells from NHP.

Supplementary Figure 3: LYPD1 TCBs demonstrate avidity-driven binding and selective T cell-mediated cytotoxicity. (a) FACS histograms demonstrate LYPD1-specific binding across all formats on the ovarian cancer cell line OVCAR3 and KURAMOCHI, with binding only limited to high affinity binders on the low-expressing cell line OVSAHO. (b) Surface plasmon resonance shows avidity driven binding of the bivalent 2+1 anti-LYPD1 TCBs as measured by slower K_{off}. (c) LYPD1-directed TCBs with high affinity

TAA induce T cell-mediated cytotoxicity by RTCC assay in LYPD1$^{medium/high}$ cell line OVCAR3 without inducing cellular killing in the LYPD1^{negative} cell line OVCAR8. (d) FACS histograms demonstrate LYPD1 cell-surface expression 2.6-fold above non-stained control in OVCAR8 cells engineered to express LYPD1. (e) LYPD1-directed TCB with high affinity TAA VHP354 induces T cell-mediated cytotoxicity by RTCC assay in OVCAR8 cells engineered to express LYPD1.

Supplementary Figure 4: Cytokine response for LYPD1-directed bivalent and monovalent TCBs. Cytokine release assays by MSD demonstrate absent IFN-γ production in the (a) LYPD1-negative cell line OVCAR8 but IFN-γ production in the (b) medium/low LYPD1-expressing cell line KURAMOCHI following 48 hour treatment with the LYPD1-directed TCBs. (c) Sequence alignment to show CD3 HuT development strategy of replacing mouse CD3ε sequence with human SP34 epitope and functional characterization by PCR and depletion of B-cells via CD20xCD3 TCB at 1mg/kg. (d) Magnitudes of change for IFN-γ, IL-6 and IL-1β from CD3 human transgenic mice treated with LYPD1-directed TCBs were unchanged at 6 h and 24 h post dose. (e) Histopathology shows no evidence of tissue destruction across LYPD1-expressing tissues pituitary, pancreas, cerebrum, and testes. Scale bar represents 200 um. (f) IL-6, TNFα and IL-1β levels were unchanged across dose groups as demonstrated by RNA-ISH. Scale bar 30 um.

Supplementary Figure 5: Toxicokinetics of VHP354 and QZC131 in cynomologus monkeys

(a) Histopathology shows no evidence of tissue destruction across LYPD1-expressing tissues pancreas, pituitary, liver and colon. (b) Treatment with LYPD1-directed TCBs VHP354 and QZC131 demonstrated modest increases in serum IFN-γ at 6 hours post-dose in most animals with partial to complete resolution by 24 hours post-dose. Cytokine changes by greater than 2-fold were considered significant. (c) IHC of CD3+ infiltrates demonstrated increased CD3+ cells in the pancreas tissue across all dose groups without a change in IFN-γ levels. Scale bar represents 30 um. (d) PK profiles of QZC131 and VHP354 demonstrated rapid clearance with an overall mean of 0.790 ± 0.133 mL/h/kg for QZC131 and 1.44

mL/h/kg for VHP354, 1.8-fold greater than the low affinity QZC131. Clearance values were calculated as Dose / AUC_{∞}. (e) RTCC assay with $LYPD1^{negative}$ cell line OVCAR8 demonstrates no induction of T cell-mediated cytotoxicity for VHP354 and QZC131 following 0, 6, 24, and 168 hrs incubation in cynomolgus serum at 37°C. (f) LYPD1-directed TCBs VHP354 (red) and QZC131 (blue) demonstrate selective and dose-dependent induction of T cell-mediated cytotoxicity for the DMS273-LYPD1-expressing cell line. 10-point dose response curves from a starting concentration of 30 nM demonstrate activity differentials across TCBs. (g) RTCC assays with $LYPD1^{negative}$ cell line OVCAR8 show no induction of T cell-mediated cytotoxicity from animal P0201 serum for VHP354 (0.1 mg/kg) at 0, 0.5 and 24 hrs post-dose and from animals P0101 (3 mg/kg) for QZC131 (3 mg/kg) at 0, 0.5, 24, 72 and 168 hrs post-dose.

Supplementary Figure 6: Dose-dependent tumor growth inhibition in AdT in vivo model

(a) LYPD1 cell surface expression is shown by FACS for the cell lines U251 ($LYPD1^{high}$) and DMS273-LYPD1 ($LYPD1^{high}$). (b) Ablation of LYPD1 cell surface expression by CRISPR-CAS9 in U251 shown by FACS. (c) LYPD1-directed TCBs with high affinity 1+1 TAA and low affinity 2+1 TAA show limited T cell-mediated cytotoxicity by RTCC assay at concentrations above 30nM in U251 cells with CRISPR knock-out (KO) of LYPD1. (d) Body weight measurements indicated no weight loss for the duration of the study across all dose groups. (n=5) (e-g) In vivo xenograft ADT model demonstrates LYPD1-directed TCBs VHP354, QWG430 and QZC131 induce dose-dependent tumor growth inhibition at 3mg/kg (black), 0.3mg/kg (orange), 0.03mg/kg (green) compared to isotype control (brown) (n=5).
